# Multifunctional Nanomaterials for the Carbon Dioxide Geological Utilization and Storage: Applications and Technical Challenges

**DOI:** 10.3390/nano16211359

**Published:** 2026-10-25

**Authors:** Shen Mao, Pengwei Fang, Yan Zhu, Qun Zhang, Meiwen Cao

**Affiliations:** 1 College of Engineering Science, University of Chinese Academy of Sciences, Beijing 100049, China; maoshen24@mails.ucas.ac.cn (S.M.); fangpengwei21@mails.ucas.ac.cn (P.F.); zhuyan23@mails.ucas.ac.cn (Y.Z.); 2 Institute of Porous Flow and Fluid Mechanics, University of Chinese Academy of Sciences, Langfang 065007, China; 3 State Key Laboratory of Enhanced Oil and Gas Recovery, Beijing 100083, China; 4 Research Institute of Petroleum Exploration & Development, PetroChina, Beijing 100083, China; 5 Laboratory of Reservoir Physics and Fluid Mechanics, China National Petroleum Corporation, Langfang 065007, China; 6 State Key Laboratory of Heavy Oil Processing and Department of Biological and Energy Chemical Engineering, College of Chemical Engineering, China University of Petroleum (East China), Qingdao 266580, China

**Keywords:** nanomaterials, CCUS, CGUS, enhanced oil recovery, CO_2_-EOR

## Abstract

As global warming intensifies, carbon dioxide (CO_2_) has become one of the primary contributors to climate change. Carbon capture, utilization, and storage (CCUS) technology represents one of the most effective methods for reducing CO_2_ emissions, offering a promising solution by capturing CO_2_ and either utilizing it or storing it in underground reservoirs for long-term sequestration. Among various CCUS strategies, CO_2_ geological utilization and storage (CGUS), which focuses on the geological aspects of CO_2_ storage and its potential utilization, plays a pivotal role. However, existing CGUS technologies still face challenges, including high costs and low efficiency. To enhance CGUS efficiency, the development of innovative technologies and materials is essential for improving the overall capability of the processes. Nanomaterials are increasingly recognized as an ideal solution for enhancing CGUS technology due to their ultra-small size, high specific surface area, exceptional physical and chemical properties, and potential for functionalization. This review examines the latest advancements in the application of nanomaterials for CGUS, focusing on the characteristics and potential of metal oxide nanomaterials, carbon-based nanomaterials, and inorganic nanomaterials, particularly silicon dioxide (SiO_2_). It also addresses the technical challenges that hinder the practical implementation of nanomaterials, including issues related to stability, scalable production, and environmental risks. Furthermore, the article outlines key research challenges and future directions for advancing CGUS technologies. The aim of this review is to provide a theoretical foundation and technical insights to drive innovation in CGUS technology.

## 1. Introduction

Rising atmospheric carbon dioxide (CO_2_) concentrations, primarily driven by anthropogenic emissions, are a major driver of global warming and climate change. Currently, atmospheric CO_2_ concentrations have surpassed 410 ppm, marking a significant increase from the pre-industrial level of 280 ppm [1,2]. To effectively combat climate change, it is essential to drastically reduce greenhouse gas emissions, particularly CO_2_. In response, extensive global research has focused on enhancing CO_2_ capture efficiency. The primary carbon capture technologies currently in use include absorption, membrane separation, cryogenic separation, and chemical cycling methods [3]. However, the high costs associated with these technologies remain a significant barrier to their widespread applications. Among various strategies for emission reduction, carbon capture, utilization, and storage (CCUS) stands out as a promising technology with the potential to substantially reduce greenhouse gas emissions [4]. As a key component of CCUS, carbon dioxide geological utilization and storage (CGUS) technology not only captures CO_2_ for underground storage to prevent direct atmospheric emissions but also enables efficient utilization of CO_2_ during injection, providing both economic and environmental benefits [5,6]. Despite advancements made in laboratory and pilot stages, CGUS still faces significant challenges in large-scale applications, including poor CO_2_ mobility control, early gas breakthrough, reservoir heterogeneity, uncertain long-term storage security, slow mineralization kinetics, leakage risks, and high operational costs.

The engineering viability of CGUS is not dictated by the ability of an individual injection well to accept carbon dioxide or a CO_2_-rich injection fluid at the required rate within its permitted pressure limits. Instead, it hinges on maintaining a dynamic balance among three tightly coupled objectives: scale storage security, CO_2_ utilization efficiency, and the timeliness of mineralization-driven solidification. These constraints are neither independent nor linearly additive. Rather, they interact within the same subsurface system, continuously counterbalancing one another across spatial and temporal scales. First, achieving higher injection rates of CO_2_ or CO_2_-rich fluids into deep saline aquifers, depleted oil and gas reservoirs, and CO_2_-EOR target reservoirs, together with larger sweep volumes, typically requires increasing the pressure differential and reshaping fluid migration pathways. Such operational intensification can induce pronounced pressure perturbations, phase evolution, and multiscale nonlinear flow behavior, thereby elevating risks associated with wellbore integrity degradation, fracture and fault reactivation, and caprock or interface leakage [7,8]. Second, accelerating mineralization can enhance permanent CO_2_ immobilization and reduce long-term leakage risk. However, if mineral precipitation occurs too rapidly near the injection well, carbonate or other secondary minerals may accumulate on pore surfaces and within pore throats, forming near-wellbore reaction-induced scale. This scaling is typically caused by reactions between injected CO_2_ or CO_2_-rich brine, formation water, and reservoir minerals, which release or concentrate ions such as Ca^2+^ and Mg^2+^ and promote carbonate precipitation. Such near-wellbore deposits are important because they can clog pore throats, reduce local permeability, increase injection pressure, impair sustained CO_2_ injectivity, and ultimately constrain scalable CGUS deployment [5,7]. Furthermore, CGUS performance is highly sensitive to wettability alteration, capillary-pressure hysteresis, relative-permeability path dependence, and pore-structure heterogeneity, which collectively introduce strong time-dependent uncertainty in multiphase transport and trapping mechanisms (for example, residual trapping) thereby further complicating engineering design and risk governance.

At the scale-up deployment stage, several rate-limiting physicochemical mechanisms continue to constrain the engineering scalability and risk controllability of CGUS. First, the pronounced mobility contrast between CO_2_ and formation brine (or crude oil in CO_2_-EOR) readily triggers viscous fingering and gravity override, causing early gas breakthrough and poor macroscopic sweep efficiency [9]. Injected CO_2_ as it migrates through reservoir pores, can develop an irregular and spatially heterogeneous morphology due to flow instabilities, which substantially increases the difficulty of closed-loop monitoring and operational control [10]. Second, multiphase interfacial behavior under reservoir temperature and pressure conditions is governed by coupled changes in wettability, interfacial tension, capillary pressure, and relative permeability; together with formation heterogeneity, these changes can promote dominant flow channels, aggravate uneven CO_2_ migration, and increase the risk of CO_2_ escape and leakage [11]. Furthermore, the kinetics of mineral sequestration impose significant limitations. Although mineral carbonation represents the most thermodynamically stable end state of sequestration, its rate is often constrained by interphase mass-transfer efficiency, reactive surface availability, and the supply of relevant ions from formation water, rendering the process too slow to satisfy practical engineering timescales [12]. Finally, the compatibility and economic viability of chemical additives remain substantial practical barriers to field-scale CGUS deployment, many existing surfactants, polymers, suffer from inadequate thermal stability, strong adsorption/retention, shear degradation, and susceptibility to shear degradation, while also exhibiting poor tolerance to divalent ions (e.g., Ca^2+^/Mg^2+^) present in formation brines under high-temperature and high-salinity conditions. These deficiencies not only erode the persistence of mobility control and storage enhancement, but also drive-up chemical dosage and life-cycle operating costs, ultimately compromising project economics and the replicability of engineering solutions [13,14].

In this context, nanomaterials solid particles with at least one characteristic dimension in the 1–100 nm range offer an emerging materials platform to address several of the above physicochemical and engineering limitations in CGUS. Depending on their geometric dimensionality, they are commonly categorized as zero-dimensional (0D; e.g., nanoparticles and quantum dots), one-dimensional (1D; e.g., carbon nanotubes and nanowires), two-dimensional (2D; e.g., graphene and nanosheets), and three-dimensional (3D; e.g., aerogels and nanocrystalline bulk architectures) [7]. Their shared attributes strong size-dependent effects, exceptionally high specific surface area, and elevated surface energy enable distinctive advantages in interfacial regulation, mass-transfer intensification, and reaction promotion. With the rapid maturation of nanotechnology, the ability to tailor surface chemistry and tune interfacial behavior has opened new technical pathways for mitigating multiphase transport instabilities, suppressing interfacial runaway and channeling, and enhancing mineralization efficiency in CGUS [15,16].

Building on their microscale effects and engineerable surface characteristics, nanomaterials have been widely recognized as promising functional candidates for improving CGUS performance [17,18]. With the maturation of this research field, it has become increasingly evident that modifying nanoparticle surfaces through functional-group grafting, polymer coating, or surface-charge regulation can markedly improve their dispersion, retention, and interfacial activity in porous media. At the pore scale, nanomaterials can reconfigure rock-fluid interfacial properties, enabling wettability alteration and interfacial-tension reduction. These changes, in turn, strengthen capillary trapping and contribute to enhanced storage security. In terms of flow control, nanomaterials can act as robust stabilizers for foams and emulsions, suppressing viscous fingering, improving macroscopic sweep efficiency, and delaying gas breakthrough, thereby promoting a more favorable balance between CO_2_ utilization and geological storage [16]. This establishes a better dynamic equilibrium between resource utilization and geological storage. Moreover, reactive or catalytic nanomaterials may accelerate carbonate precipitation and mineralization by supplying heterogeneous nucleation sites, regulating local pH and ionic strength, and modifying interfacial mass-transfer conditions offering additional pathways toward durable, long-term sequestration [19].

Notwithstanding these advantages, the field-scale adoption of nanomaterials is still impeded by multiple engineering bottlenecks that directly affect performance persistence, operational safety, and cost-effectiveness. First, thermodynamic and colloidal stability of nanomaterial dispersions in aqueous injection media, particularly high-salinity formation brines or CO_2_-saturated brines, remains a central concern. Under high-temperature and high-salinity reservoir conditions, nanomaterials introduced as aqueous nanofluid suspensions or as stabilizers in CO_2_ foam systems may aggregate in the bulk injection fluid, at CO_2_-brine interfaces, or within reservoir pore throats after entering porous media. This aggregation is promoted by charge screening, partial dissolution, coordination exchange, and surface chemical restructuring, which can weaken targeted wettability control and interfacial-tension reduction over time and space [20,21]. Second, nanoparticle transport in porous media is governed by a coupled set of mechanisms, including deposition and adsorption, mechanical filtration, bridging and pore-throat blockage, and shear-induced aggregation. While moderate, controllable retention may be beneficial for profile control and preferential-pathway management, uncontrolled retention can impair permeability and damage the near-wellbore zone, driving up injection pressures, reducing injectivity, and potentially elevating operational risks [22]. Third, systematic evaluations remain insufficient regarding the ultimate fate of nanomaterials in complex subsurface environments, their potential ecotoxicological impacts in the event of leakage, and their life-cycle performance and sustainability [23]. Finally, the lack of standardized experimental protocols and benchmarking metrics makes it difficult to directly compare results reported by different studies. This problem arises because experimental conditions often vary widely, including temperature and pressure, brine salinity, core type, fluid composition, test scale, and characterization methods [24,25]. Coupled with the scarcity of field-scale demonstrations, these gaps hinder rigorous validation of laboratory-derived conclusions and impede robust assessment of transferability and techno-economic feasibility under realistic geological conditions.

In summary, despite the promising potential of nanomaterials in CGUS, their large-scale implementation still faces several significant challenges. These include the optimization of nanoparticle dispersion, cost-effectiveness, scalability of applications, and potential environmental impacts. A thorough understanding of these challenges, along with the development of effective solutions, is essential for advancing the widespread adoption of nanomaterial-based technologies in CGUS. This paper aims to provide a comprehensive understanding of the applications and future prospects of nanomaterials in CGUS. It also critically reviews the properties of various types of nanomaterials and their specific roles in enhancing CO_2_ storage and utilization processes.

## 2. Principles and Methods of CGUS

### 2.1. Fundamental Principles of Chemically Assisted CO_2_ Geological Storage

CO_2_ geological storage involves the injection of captured CO_2_ into deep underground formations to reduce atmospheric CO_2_ concentrations. This process typically occurs at depths exceeding 800 m, where temperature and pressure conditions maintain CO_2_ in a supercritical state, thereby optimizing storage efficiency by minimizing volume. The core principle of CO_2_ geological storage involves injecting CO_2_ into deep porous reservoir rocks, where CO_2_ occupies interconnected pores and pore throats located between mineral grains within the rock matrix [26,27]. Depleted oil and gas reservoirs, deep saline aquifers, and unminable coal seams are considered suitable geological formations for CO_2_ storage [28]. Deep saline aquifers offer the largest potential storage capacity globally. Each geological environment presents unique advantages and challenges for CO_2_ storage [29,30]. In geological storage, CO_2_ is sequestered through various capture mechanisms that operate on different timescales, working in tandem to ensure the safe and secure storage of CO_2_. A comprehensive understanding of these mechanisms is essential to further optimize CO_2_ sequestration, improving its long-term stability and safety.

#### 2.1.1. Structural Trapping Mechanism

**Figure 1:**
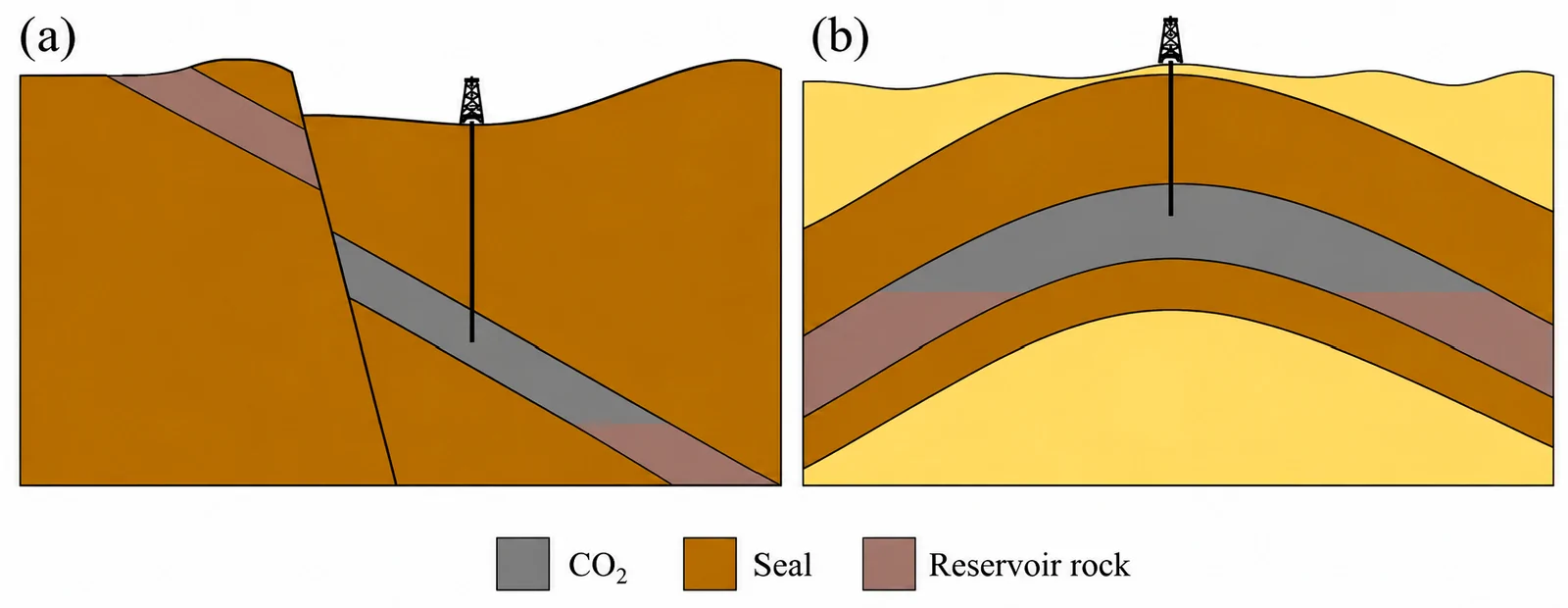
Cases of structural trapping of CO_2_: (**a**) by a subsurface fault structure and (**b**) by an overlying impermeable seal rock in a fold system [30]. Copyright 2024 Elsevier.

Structural trapping involves utilizing the physical properties of underground geological formations to securely sequester injected CO_2_, thereby preventing its migration to the surface. As shown in Figure 1, this process relies on the density contrast between CO_2_ and surrounding fluids, such as water or oil. Attributable to CO_2_’s lower density, it naturally rises under buoyant forces until it encounters an impermeable cap rock. Typically composed of low-permeability materials like clay or rock salt, the cap rock effectively isolates CO_2_, preventing its leakage and ensuring long-term, stable storage. This mechanism acts as a crucial factor in maintaining the integrity and safety of CO_2_ storage over extended periods [31,32,33]. However, the effectiveness of structural trapping is largely contingent upon the integrity and continuity of the cap rock. Cracks or voids in the cap rock can provide pathways for CO_2_ escape, compromising the storage’s effectiveness. Therefore, enhancing the sealing properties of the cap layer is crucial for improving the success of structural trapping. Recent studies have shown that nanomaterials can be introduced into CO_2_ storage systems as stable aqueous nanofluid slugs, After entering the porous formation, these nanomaterials may be retained by adsorption, mechanical filtration, or in situ deposition, allowing them to partially fill pores or form nanoscale barriers that reduce gas or water permeability and improve sealing performance [34]. With their large specific surface area and unique chemical reactivity, these nanomaterials can occupy larger pores within the reservoir, significantly reducing water or gas permeability. This in turn strengthens the reservoir’s sealing capabilities. Moreover, by modifying the microstructure of the reservoir rock and increasing pore surface area, nanomaterials can further enhance the stability of CO_2_ storage within the reservoir [35]. Given these attributes, nanomaterials are increasingly regarded as promising candidates for enhancing the long-term stability and safety of carbon dioxide storage technologies.

#### 2.1.2. Dissolution Trapping Mechanism

**Figure 2:**
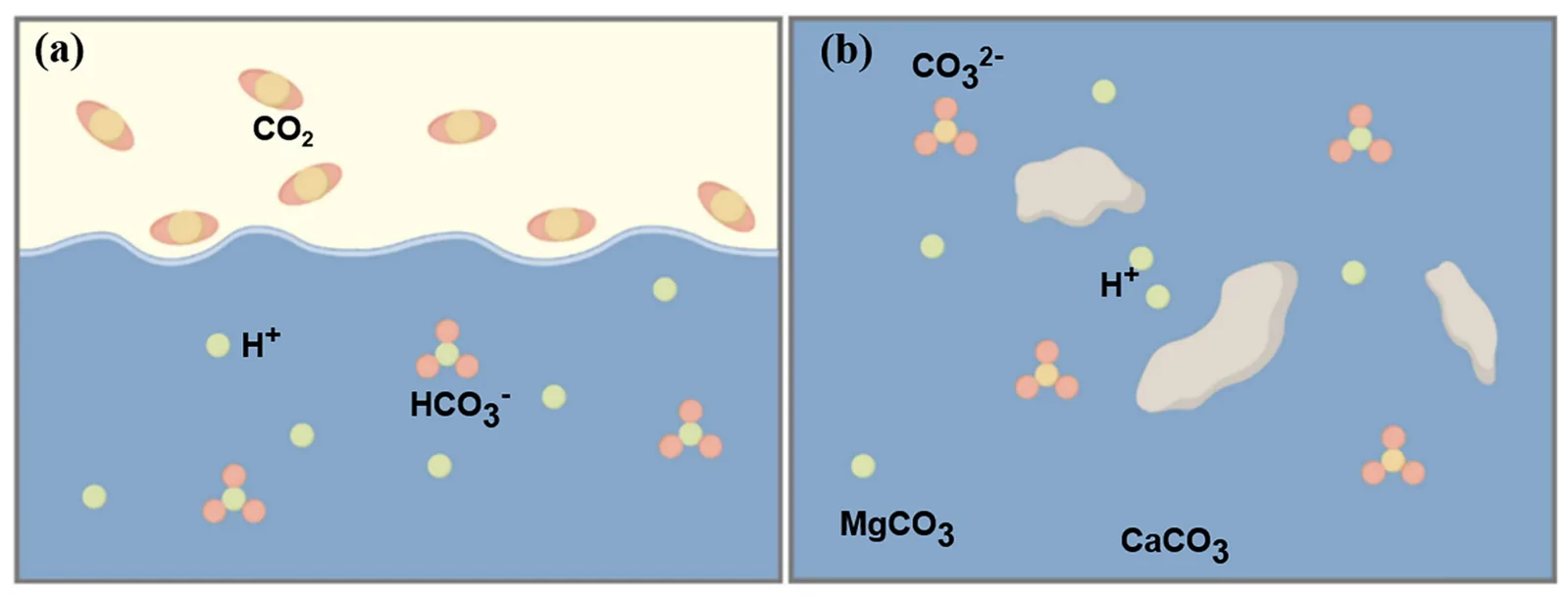
Schematic diagram of the mechanism of (**a**) solubility trapping and (**b**) mineral trapping [35]. Copyright 2025 Elsevier.

The dissolution trapping mechanism refers to the injected CO_2_ migrates upward within an aquifer driven by its lower density relative to the surrounding brine and comes into contact with formation water; the resulting dissolution produces aqueous-phase species, thereby reducing the volume of mobile, free-phase CO_2_ and lowering the risk of long-term leakage (Figure 2a). Upon interacting with water, CO_2_ is converted into a liquid form, thus reducing the risk of CO_2_ leakage during long-term storage [17,36]. CO_2_ dissolved in water demonstrates high stability, significantly lowering the likelihood of gaseous CO_2_ rising or escaping from the storage reservoir. During this process, CO_2_ enters the groundwater in its gaseous state and gradually dissolves, forming carbonic acid (H_2_CO_3_) and its dissociation products, such as HCO_3_^−^. This reaction helps stabilize CO_2_ underground by converting mobile free-phase CO_2_ into dissolved aqueous species, thereby retaining it in the formation water over storage-relevant timescales ranging from years to centuries, depending on reservoir pressure, temperature, salinity, brine mixing, and groundwater flow conditions. Its compatibility with sealing rock formations is generally favorable, and it does not depend on specific rock–mineral reactions. Moreover, this mechanism exhibits strong geological adaptability and can be applied across a variety of geological environments. One of the main advantages of dissolution trapping is the rapid nature of the reaction, particularly in the early stages following CO_2_ injection. CO_2_ dissolves quickly in groundwater, significantly mitigating the risk of gas migration. However, dissolution trapping also has certain limitations. The solubility of CO_2_ is influenced by factors such as water temperature, salinity, and pressure. Groundwater systems with high salinity or low temperatures are generally less favorable for CO_2_ dissolution, potentially leading to reduced capture efficiency. Additionally, while dissolution trapping can initially sequester significant amounts of CO_2_, its capacity decreases as the dissolved CO_2_ in groundwater gradually reaches saturation. To address these limitations, the introduction of nanomaterials presents a promising solution. Water is essential in dissolution trapping because it serves as both the solvent and the reaction medium for injected CO_2_. After CO_2_ contacts formation water or brine, it dissolves and forms carbonic acid and bicarbonate species, thereby converting mobile free-phase CO_2_ into a less mobile aqueous form. To enhance this process, nanomaterials can be pre-dispersed in formation brine or synthetic brine to form stable aqueous nanofluid suspensions, which are then injected into the reservoir through injection wells or co-injected with CO_2_-brine systems. Once introduced into the aqueous phase, these nanomaterials provide additional reactive surfaces and interfacial sites, promoting CO_2_–water contact, accelerating dissolution, and potentially facilitating subsequent carbonate precipitation. Furthermore, certain nanomaterials can interact with dissolved substances in water, leading to the formation of more stable carbonate minerals that sequester CO_2_ in a more durable form. By incorporating nanomaterials, the efficiency of the dissolution-trapping mechanism can be substantially improved, preventing the release of CO_2_ from the water and enhancing the long-term stability of CO_2_ sequestration.

#### 2.1.3. Mineral Trapping Mechanism

Mineral capture is a mechanism that transforms CO_2_ into a stable mineral form through chemical reactions with minerals in underground rocks, thus enabling the long-term fixation and storage of CO_2_ (Figure 2b) [37,38]. This process involves the interaction of CO_2_ with metal-rich minerals (such as calcium, magnesium, and iron) to produce carbonate minerals like calcite and dolomite, effectively reducing CO_2_ concentrations in the atmosphere. The high stability of these carbonate minerals ensures that they can remain intact over geological timescales, making mineral trapping one of the most enduring and secure methods for CO_2_ sequestration [39]. The advantage of mineral sequestration lies in its natural reaction mechanism, which can occur across various geological environments. Compared to other capture methods, the long-term stability of carbonate minerals is more reliable. However, the reaction rate of mineral sequestration is relatively slow, requiring significant time to effectively capture CO_2_. This limitation has somewhat constrained its large-scale application [40,41]. In recent years, the introduction of nanomaterials has provided innovative solutions for enhancing the mineral trapping process. Attributable to the highly reactive surfaces of nanomaterials, which offer more available reaction sites, the rate of CO_2_ reaction with minerals is significantly increased. The reaction between CO_2_ and minerals is typically slow at the macroscale. However, the incorporation of nanomaterials can effectively reduce the energy barrier of this reaction, accelerating the mineralization process. For instance, certain nanomaterials can facilitate the reaction between metal ions and CO_2_ to form stable minerals such as calcium carbonate and magnesium carbonate. While this process generally takes a considerable amount of time at the macroscale, the catalytic action of nanomaterials substantially improve the reaction rate, enabling CO_2_ to mineralize more quickly and achieve long-term storage [42,43].

### 2.2. Principles and Fundamental Methods of CO_2_ Geological Utilization

CO_2_ geological utilization refers to the injection of captured CO_2_ into underground formations through engineered methods, leveraging mechanisms such as multiphase seepage, phase behavior, and chemical reactions, while simultaneously enabling the long-term storage of CO_2_. This process not only ensures the secure storage of CO_2_ but also provides a viable pathway for its utilization. A typical application of CO_2_ geological utilization is CO_2_-enhanced oil recovery (CO_2_-EOR), which enhances oil recovery by injecting CO_2_ into the reservoir. The interaction between CO_2_ and crude oil causes the oil to expand, reducing its viscosity, and facilitating the extraction of lighter components from the crude oil [44]. Compared to other gases such as methane, nitrogen, or petroleum gas, CO_2_ exhibits stronger solubility and lower minimum miscibility pressure (MMP) under low-temperature, high-pressure conditions [45]. These characteristics make CO_2_ particularly advantageous for oil extraction, effectively improving oil field recovery rates. Common CO_2_-Enhanced Oil Recovery techniques include continuous CO_2_ flooding, CO_2_–water alternating flooding, and CO_2_ foam flooding. A deeper understanding of the interplay between these technologies and optimization strategies can help optimize CO_2_ injection and storage. By integrating various technologies, the efficiency and economic viability of CO_2_ geological utilization can be enhanced, while ensuring the safe storage of CO_2_, thereby promoting the widespread adoption of CGUS technology.

#### 2.2.1. CO_2_ Flooding

**Figure 3:**
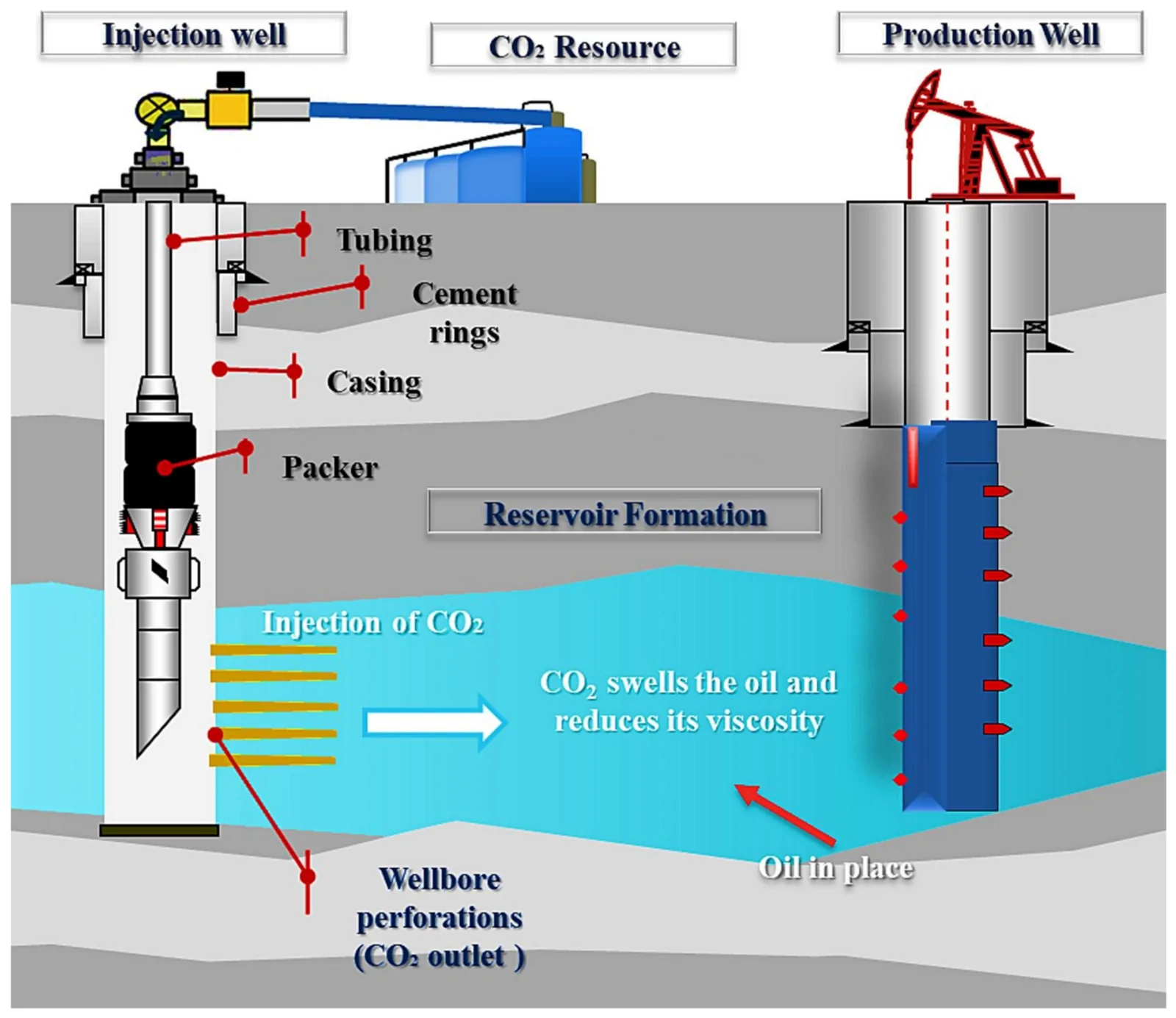
Diagrammatic representation of continuous injection of CO_2_ into an oil reservoir [27]. Copyright 2024 Elsevier.

As shown in Figure 3, CO_2_ flooding is a complex process that incorporates multiple injection techniques, involving CO_2_ capture, transportation, injection, dissolution, oil recovery, and the long-term storage of CO_2_ in reservoirs. In CO_2_-EOR, crude oil is the target phase, and its viscosity, composition, and interaction with injected CO_2_ directly determine the efficiency of oil displacement. During continuous CO_2_ injection, CO_2_ dissolves into the crude oil, causes oil swelling, extracts light hydrocarbon components, and reduces oil viscosity, thereby improving oil mobility and facilitating. This process creates an oil–gas transition zone, which gradually expands with ongoing CO_2_ injection, displacing crude oil toward the production wells. Consequently, continuous CO_2_ injection allows for long-term, sustained oil production in certain reservoirs. Additionally, CO_2_ is permanently stored over time by dissolving and becoming entrapped in pore spaces [46]. However, due to the viscosity and density differences between CO_2_ and crude oil, rapid breakthrough of CO_2_ in production wells may occur, leading to issues such as gravity segregation and viscous fingering [47,48]. The addition of nanomaterials can significantly improve CO_2_ mobility, addressing challenges particularly in complex reservoirs. As a result of their extremely small size, nanomaterials can penetrate micro-pores and fine fractures within the reservoir. By diffusing throughout the reservoir and interacting with oil and gas, they help CO_2_ distribute more uniformly, preventing the formation of flow fingers. Additionally, nanomaterials enhance CO_2_ permeability in rocks by physically and chemically interacting with the solid surfaces within the reservoir. This promotes deeper diffusion of CO_2_, thereby improving oil recovery efficiency [49]. Furthermore, nanomaterials can enhance the storage and retention time of CO_2_, effectively increasing the amount of CO_2_ retained in the reservoir. As a result, nanomaterials not only improve the effectiveness of CO_2_ sequestration but also enhance CO_2_ utilization efficiency, contributing to increased oil recovery and providing valuable support for CO_2_ geological storage [50].

#### 2.2.2. Water-Alternating-Gas Flooding

**Figure 4:**
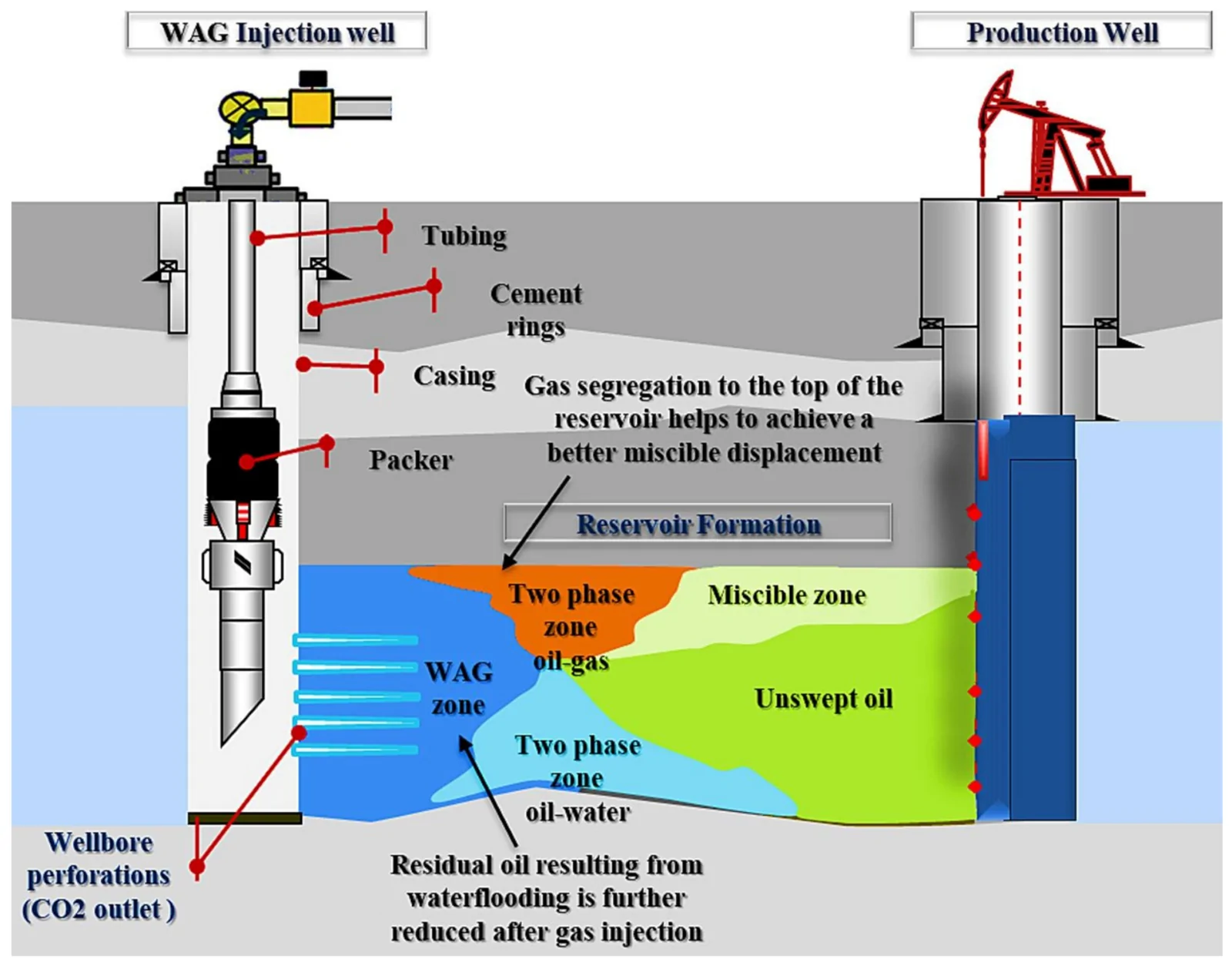
Diagrammatic representation of WAG injection into an oil reservoir [27]. Copyright 2024 Elsevier.

Water-Alternating-Gas (WAG) flooding is a technology that alternates and periodically injects CO_2_ and water into the reservoir (Figure 4). By combining the significant microscopic oil displacement efficiency of CO_2_ flooding with the macroscopic sweep efficiency of water injection, WAG optimizes oil recovery. The underlying technique is based on the swelling effect of CO_2_ on crude oil under formation pressure, the reduction in viscosity, and the lowering of interfacial tension, all of which enhance oil production levels in a single well [33,51]. During the water injection stage, the relatively low mobility of the water phase effectively blocks high-permeability zones and pre-existing gas channels, expanding the overall swept volume and stabilizing the displacement front [52]. This alternating injection cycle improves recovery rates without significantly increasing the total injected gas volume, thereby reducing the risk of early gas breakthrough. Compared to continuous CO_2_ injection methods, the WAG process profoundly enhances CO_2_ mobility and sweep efficiency in certain reservoirs, while mitigating issues related to viscous variation. However, WAG technology also presents several challenges, including low CO_2_ utilization rates, limited adaptability to specific reservoir conditions, increased costs, and complex surface facility requirements [53]. To address these issues, the introduction of nanomaterials has emerged as an effective optimization strategy. Nanomaterials can enhance the permeability of CO_2_ and water, facilitating a more uniform distribution within reservoir pores and preventing uneven flow. This improves the uniformity of water penetration throughout the reservoir, thereby enhancing crude oil displacement efficiency [54]. Furthermore, nanomaterials can modify the oil–water interface structure and reduce interfacial tension, effectively decreasing the adhesion between crude oil and reservoir rock. These improvements collectively enhance the oil recovery performance of CO_2_ and water injection, ultimately leading to increased oil recovery efficiency [55,56].

#### 2.2.3. Foam-Assisted CO_2_ Flooding

**Figure 5:**
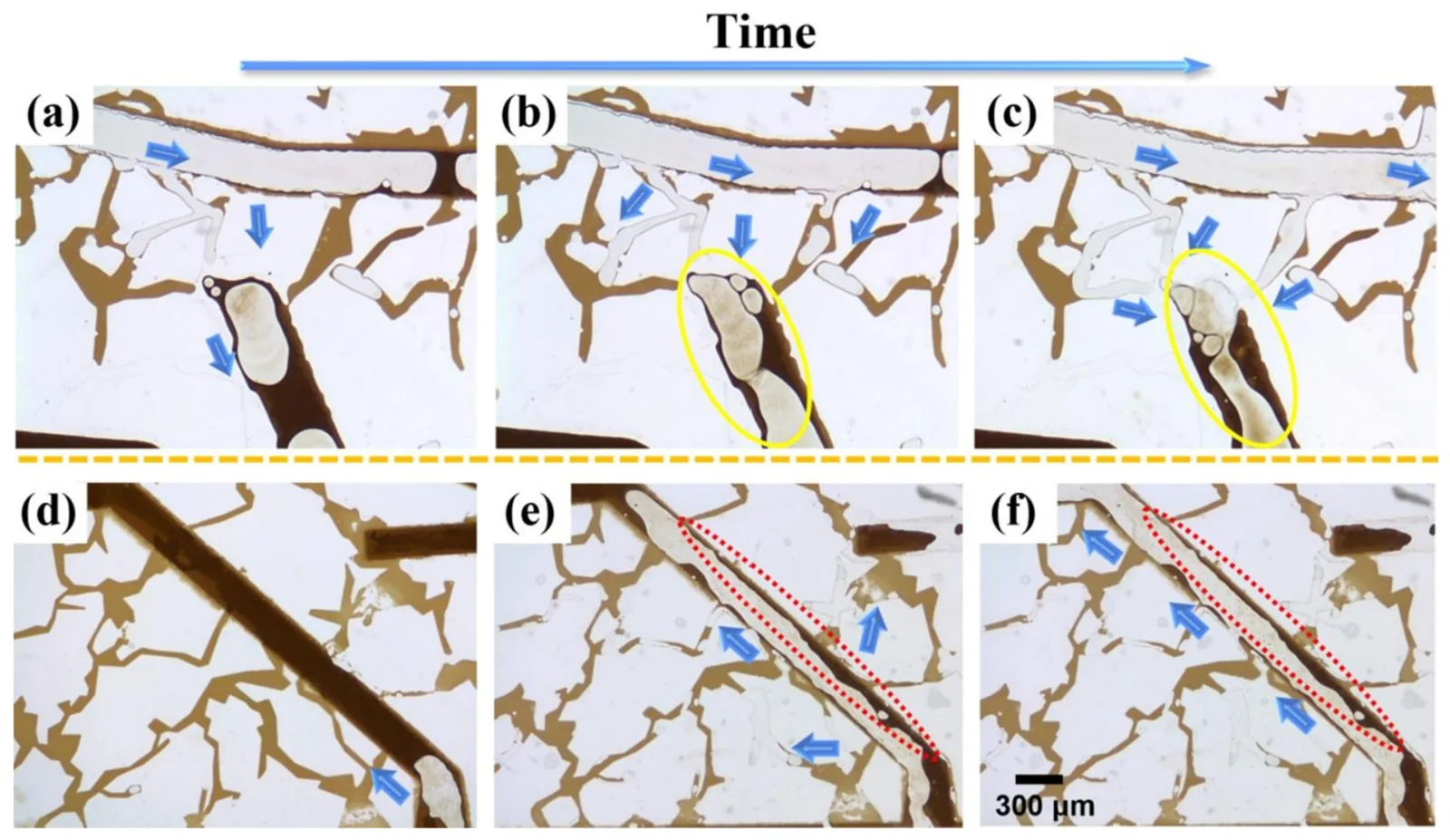
(**a**–**c**) Formation of CO_2_ blockage in fractures, and (**d**–**f**) formation of CO_2_ channel in large fractures. The yellow circle represents the merging of oil bubbles, and the red circle represents the membranous residual oil [24]. Copyright 2022 Elsevier.

Foam exhibits high apparent viscosity and favorable flow characteristics in porous media, making it a promising method for enhancing CO_2_ storage capacity [57]. CO_2_ foam systems effectively control gas-phase mobility by increasing the apparent viscosity of the gas phase during reservoir gas flooding, thereby suppressing gas migration, reducing gas channeling, and improving sweep efficiency [58,59,60]. By increasing mobility ratios and reducing oil–water interfacial tension, foam can dramatically increase sweep efficiency and facilitate the geological storage of CO_2_ [61,62,63]. However, foam is inherently a thermodynamically unstable system, and its strength and stability serve as a key component in the effective storage and utilization of CO_2_ [22,64,65]. Research indicates that nanomaterials can stabilize CO_2_ foam, withstand harsh reservoir conditions, and effectively suppress CO_2_ gas migration and the progression of viscosity fingers [66,67]. The nanomaterials possess strong pore-blocking properties, which can significantly hinder gas migration and promote the retention and storage of CO_2_ in porous media [13]. Nanoparticles are typically adsorbed at the CO_2_–water interface during bubble formation, thereby strengthening the foam films and improving foam stability. Within large fractures, stable foam can form a robust plug, facilitating foam migration into mesopores and micropores (Figure 5). This promotes the uniform distribution of CO_2_ bubbles within the fractures and enhances the utilization of gas storage space in mesopores and micropores. Additionally, CO_2_ foam stabilized by nanomaterials improves fluid dynamics, enabling the formation of uniform flow within the reservoir. This, in turn, enhances the driving effect of CO_2_ in the reservoir and facilitates smoother oil flow toward production wells [68]. Furthermore, foam can enhance the desorption and migration of oil droplets through interactions with the rock surface and oil, reducing the viscosity between oil and water and further promoting oil migration. This leads to a higher oil recovery rate [69,70].

## 3. Synthesis Methods and Advantages and Disadvantages of Different Types of Nanomaterials

In the CGUS, nanomaterials demonstrate tremendous application promise attributable to their unique physicochemical properties, particularly their high surface area, outstanding catalytic capability, and excellent stability [71]. However, nanomaterials may encounter problems such as crystal defects, incomplete structure or impurity doping during the synthesis process. These defects not only affect their physical and chemical properties but may also lead to instability under extreme conditions such as high temperature and pressure, thereby impacting the efficiency of CO_2_ adsorption, conversion, and storage [72]. To address the aforementioned issues, modifying nanoparticle surfaces by physical or chemical treatment has emerged as a viable solution. These modification methods enhance nanomaterials stability and surface activity while imparting new physical, chemical, and mechanical properties [73]. For instance, by introducing hydrophilic or lipophilic functional groups, the surface affinity of materials can be adjusted, thereby enhancing their selectivity and stability. Additionally, surface modification can enhance the corrosion resistance and high-temperature tolerance of nanomaterials. It is important to note that nanoparticle modification typically involves the combined use of multiple methods rather than relying on a single approach [74]. Thus, a rational modification strategy should be designed based on the characteristics of the chosen modifier and the desired modification outcomes. This article reviews several commonly used methods for nanoparticle surface modification. Table 1 summarizes the advantages and disadvantages of various modification methods.

### 3.1. Theoretical Basis of Dispersion Stability

For nanomaterial-enabled CGUS, dispersion stability is critical because nanoparticles must remain sufficiently dispersed during preparation, injection, and migration through porous media. Poor stability can lead to aggregation in the injection fluid or within pore throats, causing particle retention, permeability reduction, and loss of functional performance. The instability of nanoparticles mainly arises from their high surface free energy and strong interparticle attractions, including van der Waals forces and ion-mediated aggregation [75,76]. These effects become more severe in high-salinity formation brines, where electrostatic repulsion is weakened by compression of the electrical double layer.

From a microscopic perspective, the agglomeration behavior of nanomaterials is fundamentally attributed to the thermodynamic driving force of the system seeking to minimize Gibbs free energy, coupled with kinetic instability caused by insufficient repulsive potential barriers between particles.

Surface modification improves dispersion stability through two main mechanisms. First, it reduces the interfacial free energy between nanoparticles and the surrounding aqueous phase by introducing compatible hydrophilic, charged, or amphiphilic groups. Second, it increases the kinetic barrier against aggregation by strengthening electrostatic repulsion, steric hindrance, or hydration-layer protection [77]. These mechanisms are directly relevant to CGUS because stable nanofluids are more likely to maintain injectivity, migrate controllably through reservoir pores, and preserve their intended functions in wettability regulation, interfacial-tension reduction, foam stabilization, and mineralization promotion [78,79,80,81].

### 3.2. Common Methods for Modifying Nanomaterials

#### 3.2.1. Physical Methods

Physical surface modification of nanomaterials is primarily achieved through surface deposition techniques such as adsorption and coating. The functional groups on the surface of the nanomaterials interact with the modifiers through van der Waals forces, hydrogen bonds, or coordination effects, without involving ionic or covalent bonds. This method enables surface physical modification without altering the intrinsic chemical structure of the nanomaterials.

##### Heat Treatment and Mechanical Modification

To effectively modify the surface of nanomaterials and reduce particle aggregation, preliminary modification is necessary. Initial modification methods, such as heat treatment and mechanical techniques, are not only cost-effective but also easy to implement. Heat treatment reduces aggregation by breaking hydrogen bonds between nanomaterials at an optimal temperature. However, excessively high temperatures may increase the Brownian motion of nanomaterials, raising the likelihood of particle collisions [82]. Therefore, selecting a suitable temperature range during thermal treatment is essential to control nanoparticle aggregation.

**Figure 6:**
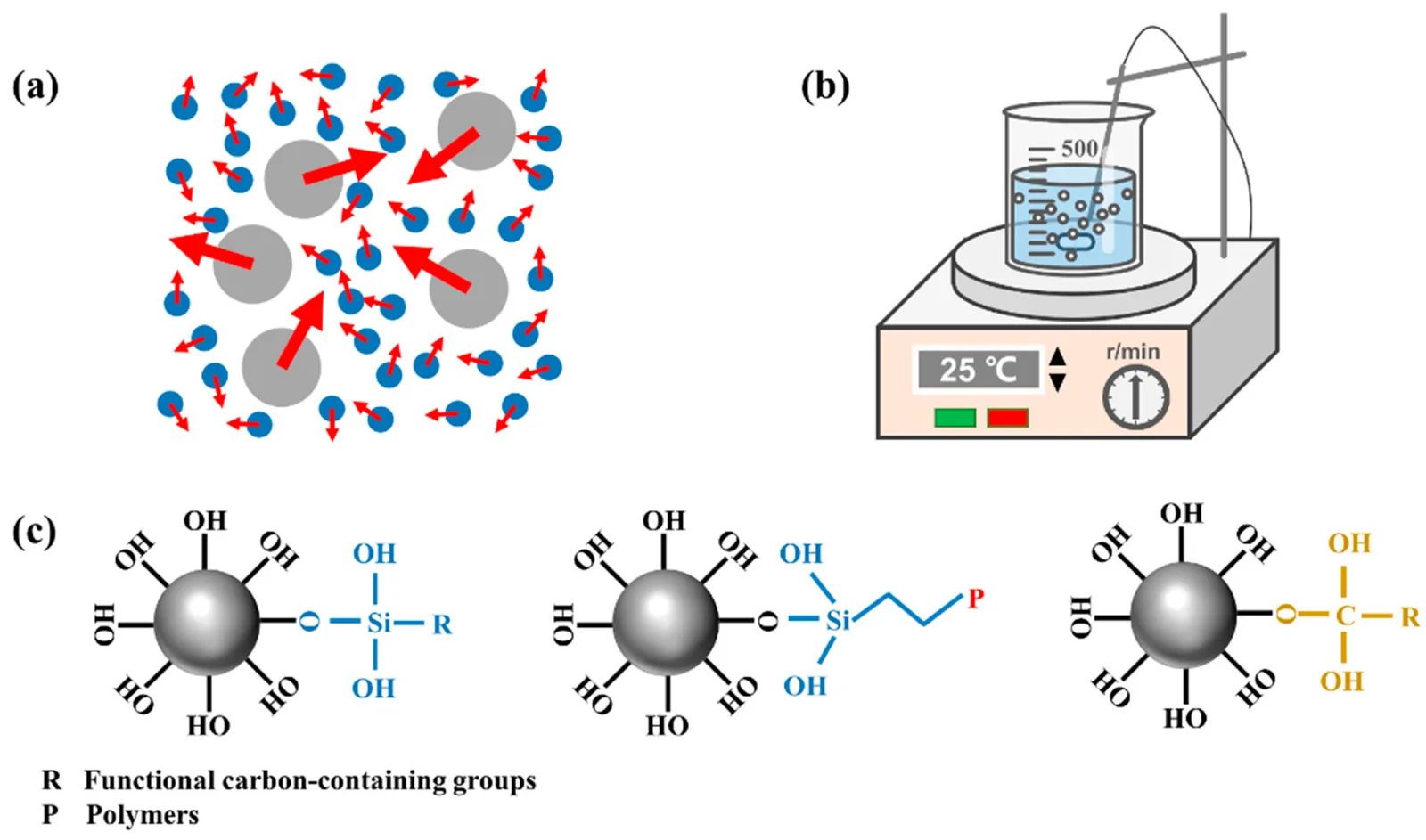
(**a**,**b**) Schematic diagram of thermal treatment and mechanical modification. (**c**) Schematic diagram of the chemical modification of nanomaterials by different chemicals (**left**: coupling modification, **mid**: surface grafting modification, **right**: esterification modification [7]). Copyright 2025 Elsevier.

Mechanical modification involves applying intense mechanical forces to nanoparticle solutions through methods such as enhanced magnetic stirring, ultrasonic agitation, high-shear mixing, and ball milling (Figure 6b). When the input mechanical energy overcomes the agglomeration potential energy barrier, the agglomerated nanomaterials redisperse. It is worth noting that although heat treatment and mechanical modification are traditionally classified as physical methods, high-energy mechanical inputs (such as ball milling) can induce a mechanical-chemical effect, in which strong shear forces break chemical bonds or activate surface lattice atoms, leading to intrinsic chemical transformations.

Overall, thermal/mechanical pretreatment can reduce agglomeration, improve particle size distribution, and enhance subsequent modification efficiency to some extent, thus laying the foundation for building a more stable nano-dispersion system.

##### Physical Adsorption Method

Physical adsorption modifies nanoparticle surfaces by exploiting non-covalent, weak interactions such as hydrogen bonding, van der Waals forces, and electrostatic attraction between nanomaterials and modifiers (e.g., surfactants, polymers). These interactions enhance colloidal stability and/or impart specific functionalities to the particles. When modifier molecules are introduced into a nanoparticle dispersion, they diffuse to the particle surface, and driven by electrostatic attraction or hydrogen bonding, assemble into an adsorbed layer of finite thickness, thereby forming a protective coating [83,84]. This method primarily stabilizes particles by manipulating the Electrostatic Repulsion and Steric Repulsion terms. Agents like Sodium Dodecyl Sulfate (SDS) introduce sulfate groups (-SO_4_^2−^), while Cetyltrimethylammonium Bromide (CTAB) introduces quaternary ammonium groups (-N^+^(CH_3_)_3_). These ionized groups significantly increase the surface charge density, thereby elevating the absolute value of the Zeta potential [7]. A higher Zeta potential creates a strong electrostatic energy barrier, effectively preventing particle collision in aqueous media. Polymers like Polyethylene Glycol (PEG) or surfactants like Tween-80 introduce hydroxyl (-OH) and ether (-O-) groups. These groups interact with water to form a robust hydration layer. Thermodynamically, this lowers the interfacial tension (γ). Kinetically, the extended polymer chains provide a finite steric layer thickness, generating steric repulsion even in the absence of charge.

##### Physical Coating Method

Physical coating is a surface modification strategy that involves embedding or encapsulating nanomaterials within another material (such as polymers, liposomes, silica, or inorganic matrices) through physical forces, including hydrophobic interactions, van der Waals forces, electrostatic attraction, or steric hindrance. This results in the formation of core–shell structures or complexes, effectively altering the surface morphology of nanomaterials and significantly enhancing their performance [85]. This modification reduces van der Waals attraction, while the thick coating introduces significant steric hindrance, forming a robust physical barrier to prevent aggregation. The coating material adheres uniformly to the surface of the nanomaterials, forming a continuous, complete monolayer coating. This modification not only imparts novel surface properties to the nanomaterials but also improves their dispersibility, stability, and interactions with the surrounding environment.

In the aforementioned methods, nanomaterials interact with modifiers through electrostatic attraction or repulsion, hydrogen bonding, van der Waals forces, and hydrophobic or hydrophilic interactions. These interactions are primarily physical in nature, relying on relatively weak forces that are highly sensitive to external environmental conditions. Variations in temperature, mineralization, and pH can significantly influence the efficacy of the modifier in altering the properties of nanomaterials.

#### 3.2.2. Chemical Methods

The large specific surface area of nanomaterials results in a higher proportion of surface atoms, which possess unpaired electrons as a result of coordination unsaturation. This leads to unpaired chemical bonds, imparting high reactivity to the surface atoms and making them highly prone to bonding with other atoms [86]. Therefore, this characteristic creates favorable conditions for the chemical modification of nanoparticle surfaces.

##### Coupling Modification

Coupling agents are a common method for the surface modification of nanomaterials and are widely utilized in the functionalization of nanomaterials. These agents are molecules containing two or more functional groups, with one end capable of chemically reacting with the functional groups on the surface of nanomaterials, while the other end can interact with organic compounds, polymers, or other functional molecules to form a functionalized interface (Figure 6c left). A typical example of a coupling agent is silane coupling agents, which are frequently used for nanoparticle surface modification. In the presence of trace amounts of water, the alkoxy groups of silanes coupling agents undergo hydrolysis, generating active silanol groups that form stable Si-O bonds with the hydroxyl groups on the nanoparticle surface. Simultaneously, the functional groups at the other end of the silane molecule (e.g., amino groups, carboxyl groups) can bond with organic matter or other substrates [84,87,88]. For example, long hydrophobic alkyl chains (-C_18_H_37_) are introduced via OTES. These long chains reduce the solid–liquid interfacial tension between nanomaterials and organic solvents, promoting their dispersion in nonpolar media. The introduction of amino groups via APTES protonates these groups to -NH_3_^+^ under acidic conditions, dramatically increasing the zeta potential and providing strong electrostatic stability. Furthermore, reactive amino groups can serve as universal anchors for further functionalization [7]. Thus, coupling agents serve not only as independent surface modifiers but also as intermediates in other modification methods, enabling more complex surface modifications.

##### Surface Grafting Modification

Surface grafting modification refers to a technique that achieves specific encapsulation effects by polymerizing organic monomers on nanoparticle surfaces or grafting polymers onto particle surfaces through polymer reactions (Figure 6c mid) [89,90]. Through chemical bonding, molecules with specific functional groups are modified onto the particle surface, endowing the particles with unique physicochemical properties that they originally lacked, such as hydrophilicity, lipophilicity, super adsorption capacity, and high-temperature catalytic resistance, At the same time, this is also the most effective way to manipulate steric repulsion by controlling polymer chain length and density [91,92]. The materials prepared through surface grafting polymerization exhibit excellent performance complementarity and synergistic effects. By grafting monomers such as acrylic acid (AA) and methyl methacrylate (MMA) to introduce functional groups such as carboxyl (-COOH) and ester groups, the zeta potential can be improved, while the extension of the polymer chain provides some steric hindrance. This dual mechanism makes the dispersion system highly stable to changes in pH and salt concentration. There are two primary methods for surface graft polymerization. The first method involves chemical bonding, where functional groups or reactive radicals are attached to the particle surface to achieve modification. This method is simple to operate, providing strong control over the grafting process, and offering a narrow molecular weight distribution and high grafting density [93]. The second method involves initiating polymerization directly on the nanoparticle surface in the presence of an initiator, enabling continuous chain growth [94]. This method adequately improves the grafting rate and reduces the influence of steric hindrance by first grafting functional groups onto the particle surface.

##### Esterification Modification

Esterification modification refers to the process of modifying the surface of nanomaterials by reacting the hydroxyl or carboxyl groups on the nanoparticle surface with long-chain fatty alcohols under acid catalysis or high-temperature dehydration conditions to form stable ester bonds (Figure 6c right) [95]. The chain length of the fatty alcohol serves as a key component in determining the wettability of the modified nanomaterials. When the number of carbon atoms in the fatty alcohol backbone exceeds 10, the hydrophobic groups grafted on the nanoparticle surface become longer, significantly enhancing the hydrophobicity of the modified nanoparticle surface [96]. Compared to silane coupling agent modification methods, the alcohol esterification method offers the advantage of lower cost. This method is straightforward, requires no complex coupling agents, and is suitable for large-scale applications. However, it also has some drawbacks. For instance, the required chemical dosage is relatively high, and the reaction conditions are demanding. Additionally, the modified product is prone to hydrolysis and exhibits poor stability, which limits its application in certain fields.

Compared to physical modification, chemical modification of nanomaterials offers more durability results. However, this approach increases the complexity of the reaction and necessitates more stringent reaction conditions.

### 3.3. Synergistic Application of Modified Nanomaterials in CGUS: From Enhancing Oil Recovery to Secure Storage

Modified nanomaterials can establish synergistic enhancement pathways across the CGUS chain through four tightly connected mechanisms adsorption/separation intensification, interfacial regulation, mobility control, and mineralization acceleration. Collectively, these effects support upstream improvements in CO_2_ capture and separation, midstream optimization of CO_2_-EOR performance and migration management, and downstream reinforcement of storage integrity while promoting long-term stable sequestration. The underlying synergy is rooted in the deliberate coupling of surface chemistry with interfacial physics: by reshaping pore-scale wettability, interfacial tension, and phase configuration, nanomaterials enable coordinated optimization at the engineering scale, simultaneously advancing higher utilization efficiency and more reliable storage security.

#### 3.3.1. Enhance Carbon Dioxide Capture and Separation

In high-efficiency CO_2_ capture and separation, surface-engineered nanomaterials play a decisive role by improving adsorption capacity and selectivity. This is particularly evident in low-temperature solid-adsorption systems, where amine-functionalized nanomaterials have been extensively explored for CO_2_ enrichment due to their high specific surface area, tunable pore architecture, and the controllability of functional-group incorporation [49,97]. The introduction of amino functionalities increases surface Lewis’s basicity and polarity, thereby strengthening acid–base interactions and chemisorption driving forces at the gas–solid interface. As a result, high adsorption affinity can be maintained even under low CO_2_ partial pressure, while discrimination against nonpolar or weakly polar components (e.g., N_2_ and CH_4_) is improved [98,99]. Beyond functionalization per se, adsorption performance is strongly dependent on how amines are configured. Beyond functionalization per se, adsorption performance is strongly dependent on how amines are configured. By selecting amine species (primary/secondary/tertiary), tailoring the loading route (physical impregnation versus covalent grafting), and regulating their spatial distribution within pores, it becomes possible to optimize site accessibility and mass-transfer pathways. Such structural–chemical tuning helps to reconcile the intrinsic trade-offs among adsorption capacity, separation selectivity, and regeneration energy demand, thereby improving the overall process efficiency and operational feasibility of capture–separation units [100].

#### 3.3.2. Interface and Wettability Regulation

Controllable regulation of the rock-fluid interface is related to the core objectives of both crude oil utilization and long-term CO_2_ sequestration. Surface-engineered nanomaterials can preferentially adsorb onto mineral surfaces and reconfigure interfacial chemistry, typically shifting wettability toward a more water-wet state while simultaneously lowering oil–water and/or gas–water interfacial tension [101]. The coupled effect of wettability alteration and interfacial-tension reduction increases the capillary number, facilitating the overcoming of capillary confinement and enabling the remobilization of residual oil, thereby improving displacement efficiency. In addition, nanofluids can form a wedge-shaped film in the solid–liquid–oil three-phase contact region, generating a structural disjoining pressure. At the pore scale, this effect promotes oil-film detachment and interfacial rearrangement, further enhancing the release of capillary-trapped oil [102].

From the perspective of containment security, CO_2_ commonly acts as a non-wetting phase in strongly water-wet caprock systems. By increasing the hydrophilicity of caprock pore throats, nanomaterial treatment can elevate the capillary entry pressure required for CO_2_ to invade water-saturated sealing formations. This strengthens capillary sealing capacity and raises the invasion threshold, thereby suppressing upward CO_2_ migration and reducing leakage risk.

#### 3.3.3. Mobility Control

Nanomaterials with near-neutral wettability (e.g., via alkyl silane functionalization) can exhibit strong affinity for the CO_2_ brine interface and adsorb irreversibly to form a mechanically robust, particle-armored interfacial layer. Such interfacial reinforcement inhibits foam-film drainage and bubble coalescence, enhancing foam strength and improving resistance to decay. In practical injection-displacement operations, nanomaterial stabilized foams markedly increase the apparent viscosity of the CO_2_ rich phase and reduce its effective mobility, thereby suppressing viscous fingering and crossflow. The resulting mobility control redirects flow into comparatively lower-permeability regions, enlarging the swept volume and improving macroscopic sweep efficiency [8,103]. Importantly, improved sweep efficiency also promotes CO_2_ retention within a larger pore volume through residual and dissolution trapping, rather than allowing rapid breakthrough along high-permeability conduits. Consequently, the same displacement process can simultaneously enhance CO_2_-EOR performance and increase effective geological storage, enabling a coupled optimization of utilization efficiency and storage security.

#### 3.3.4. Accelerated Mineralization

Mineralization-based sequestration is widely regarded as a high-security storage pathway because carbonate products represent a geochemically stable and thermodynamically favored end state. Nevertheless, its practical implementation is constrained by two interrelated engineering bottlenecks: inherently slow reaction kinetics and the operational risks associated with flow-precipitation coupling, including near-wellbore scaling, pore-throat blockage, and permeability impairment. Consistent evidence from prior studies indicates that effective mineralization acceleration hinges on three rate-controlling steps: promoting CO_2_ hydration and carbonate-system speciation, enhancing the availability and transport efficiency of divalent cations (Ca^2+^/Mg^2+^), and strengthening carbonate nucleation and subsequent crystal growth. More recently, insights from classical nucleation theory and interfacial-energy regulation have provided a clearer mechanistic basis for nanomaterial-enabled mineralization enhancement [30,33]. Specifically, nanostructured surfaces can lower the energy barrier for heterogeneous nucleation and thereby increase the nucleation rate of carbonate phases (e.g., CaCO_3_), supporting the concept of nanomaterials/nanostructures as heterogeneous nucleation templates. Engineered nano-interfaces with lattice-matched surfaces, tailored charge distributions, and specific functional sites can enhance local supersaturation utilization and increase the density of active nucleation sites, thereby accelerating mineralization without requiring a proportional increase in bulk chemical dosage [104]. Critically, mineralization enhancement must be co-optimized with long-term injectivity, ensuring that accelerated carbonate precipitation does not induce excessive pore-throat blockage, permeability loss, or near-wellbore injectivity decline. While accelerating solidification is desirable for long-term security, material selection, dosing strategy, and spatial placement should be designed to avoid premature precipitation in the near-wellbore region, where early scaling can rapidly degrade permeability and compromise injection capacity. Achieving a robust CGUS implementation therefore requires balancing mineralization efficiency against flow continuity, ensuring that enhanced trapping does not come at the expense of operational injectability.

While the aforementioned synthesis and modification strategies provide a versatile toolkit for tailoring the physicochemical properties of nanomaterials, the laboratory-scale optimization represents only the initial step toward engineering deployment. The ultimate efficacy of these nanomaterials is not determined in the synthesis flask, but by their stability and functional performance under the harsh conditions of deep subsurface reservoirs. Consequently, it is essential to bridge the gap between material design and application, shifting focus to how these engineered nanostructures specifically address the multiphase flow and mineralization challenges inherent in the CGUS process.

## 4. Mechanism- and Function-Oriented Applications of Nanomaterials in CGUS

Surface chemistries and stability mechanisms established in the previous chapter, this section examines the functional deployment of nanomaterials across the CGUS value chain. Particular emphasis is placed on engineered properties, such as wettability alteration and interfacial-tension reduction, that help address key bottlenecks in capture efficiency, mobility control, and long-term mineralization while improving storage security and resource utilization [85].

### 4.1. Enhance Carbon Dioxide Capture and Separation

In unconventional reservoirs such as coal seams and shale where nanoporous networks and organic/mineral rich surfaces dominate the occurrence and transport of CO_2_ are governed largely by physical adsorption and surface-mediated interactions. Consequently, a coupled set of variables including pressure-temperature conditions, pore/surface properties, water content, and the presence of competitively adsorbing species collectively controls capture performance and storage efficiency. Within this framework, the contributions of nanomaterials can be organized along two complementary pathways. First, by regulating gas–liquid mass transfer and tuning bulk solution physicochemical properties (e.g., viscosity, surface tension, and interfacial renewal dynamics), nanomaterials can accelerate absorption rates and reduce mass-transfer resistance. Second, by providing high specific surface area together with engineerable active sites, nanostructured sorbents enable high-capacity and potentially regenerable adsorption/reaction-based capture. When integrated with process intensification strategies (e.g., magnetic separation), these materials can also lower sorbent recovery costs, enhance cyclic stability, and improve overall process feasibility. Table 2 summarizes recent progress on nanomaterials for enhanced CO_2_ capture and separation.

With respect to liquid-phase absorption enhancement, conventional CO_2_ capture systems typically rely on water-soluble amines (e.g., monoethanolamide, ammonia) or carbonate-based solvents (e.g., hot potassium carbonate). Despite their maturity, these routes often face persistent engineering constraints, including high regeneration energy demand, solvent degradation and corrosion risks, and in certain formulations insufficient reaction kinetics. Introducing nanomaterials into the liquid phase provides a pathway to intensify absorption by modifying hydrodynamic and interfacial transport characteristics: nanomaterials can alter effective viscosity and surface tension, increase interfacial disturbance and renewal frequency, and promote microscale turbulence and boundary-layer mass transfer, thereby improving kinetic rates and the overall mass-transfer coefficient.

**Figure 7:**
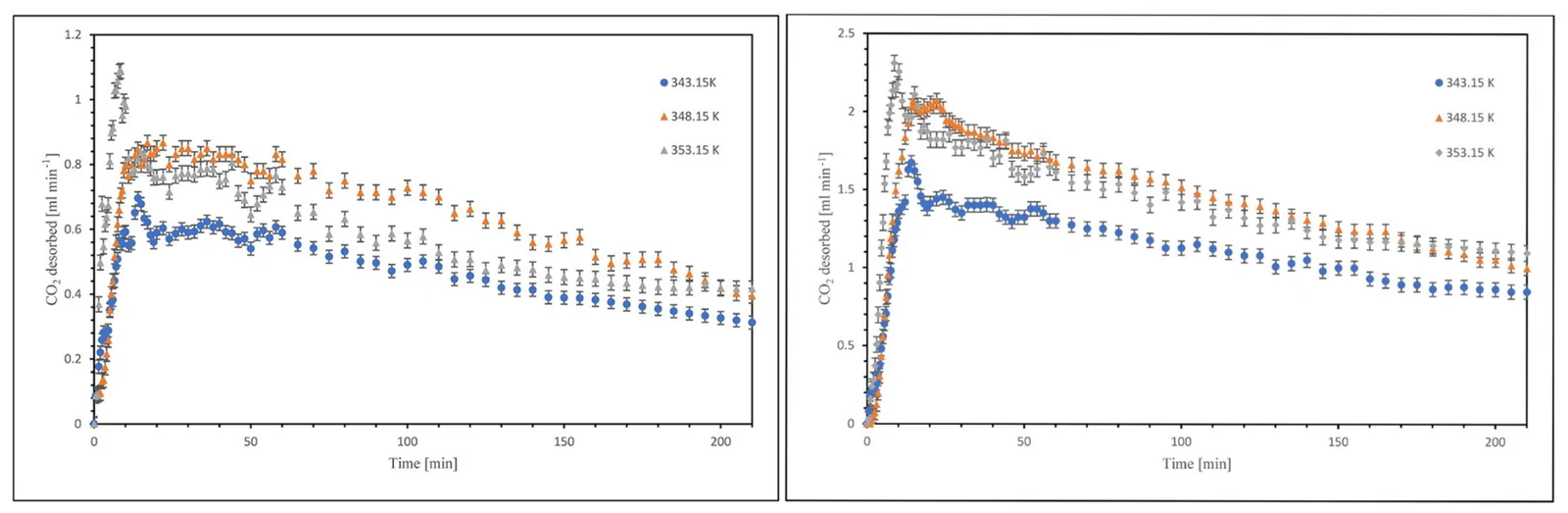
Trend of CO_2_ desorbed in the base fluid (**left**), and Trend of CO_2_ desorbed in the nanofluid (**right**) [105]. Copyright 2025 MDPI.

In recent years, a representative research paradigm has coupled nanofluid absorption experiments and kinetic/mass-transfer modeling to quantitatively relate absorption-rate enhancement to changes in transport parameters under controlled and comparable conditions. This approach is frequently complemented by spectroscopic and in situ diagnostics (e.g., infrared analysis, Raman/DSC), molecular dynamics simulations, and cyclic operation tests, enabling mechanistic interpretation of how nanoparticle interfacial behavior reshapes mass-transfer pathways and reaction rates. Such efforts are gradually establishing a transferable correlation chain linking material design mass transfer coefficient evolution absorption efficiency gains, which is essential for cross-study comparability and engineering translation. The K_2_CO_3_ system provides a representative example. Potassium carbonate is cost-effective, relatively non-corrosive, and chemically stable, and it is generally associated with lower volatility and reduced environmental burden compared with many amine-based solvents, making it attractive for large-scale CO_2_ capture. However, its practical deployment is often limited by relatively slow kinetics, which can necessitate larger equipment footprints to achieve comparable capture performance. To address this limitation, nanoparticle additives have been investigated to intensify solvent performance (Figure 7) [105]. For instance, TiO_2_ nanoparticles introduced into K_2_CO_3_ solutions have been evaluated across temperature and concentration ranges, with absorption behavior interpreted using Danckwerts theory in combination with spectroscopic evidence. The results indicate that TiO_2_ can significantly enhance absorption efficiency by tuning bulk properties and promoting interfacial mass transfer, while exhibiting an optimal operational window at specific temperatures and particle loadings [106].

Notably, these observations imply that nanoparticle-enabled intensification does not scale monotonically with dosage. Instead, the net benefit is often constrained by competing effects including dispersion stability, viscosity-induced hydrodynamic penalties, and limits on interfacial renewal leading to a window effect and/or diminishing marginal returns. Accordingly, performance evaluation should move beyond single-point, short-term absorption metrics and incorporate engineering-relevant dimensions such as cyclic evolution of mass-transfer parameters, regeneration energy requirements, solvent stability, and equipment compatibility. This broader assessment is necessary to prevent laboratory-scale instantaneous gains from becoming unsustainable under realistic, long-term operating cycles.

**Figure 8:**
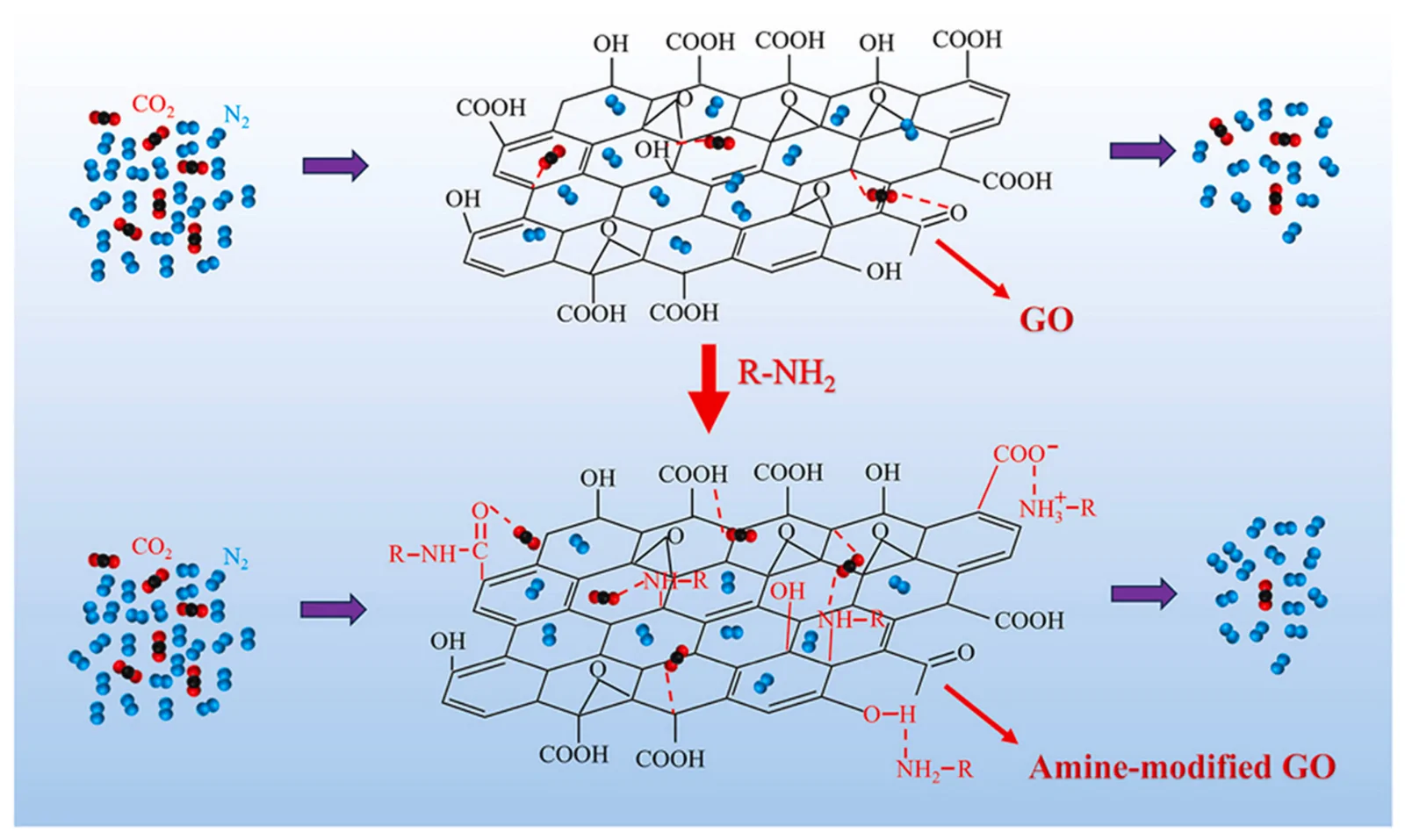
The adsorption mechanism of CO_2_ molecules by amine-modified GO [108]. Copyright 2025 Elsevier.

Nanomaterials provide system-level benefits not only by increasing CO_2_ uptake during capture but also by facilitating CO_2_ separation from mixed streams and promoting sorbent regeneration for repeated capture cycles. By integrating high specific surface area with engineerable active sites, nanostructured sorbents can simultaneously deliver high-capacity capture and robust cyclic renewability. For instance, Liu et al. functionalized graphene oxide (GO) with tetraethylenepentamine (TEPA), achieving markedly improved CO_2_ uptake and enhanced adsorption desorption stability over repeated cycles [107]. Extending this approach, Deng et al. systematically tuned GO using a suite of amine-containing modifiers, elucidating the key reaction pathways between amine functionalities and oxygenated groups on GO, and further indicating that increased amine density and stronger polar motifs can synergistically strengthen CO_2_ affinity and separation performance (Figure 8) [108].

Beyond material-level optimization, intensification of the nanomaterial-assisted CO_2_ capture process, especially the CO_2_ separation and sorbent regeneration steps, can reduce energy and mass-transfer penalties while improving tolerance to variable operating conditions. In particular, magnetically recoverable nano-sorbents can couple high-capacity capture with rapid, low-energy solid–liquid separation, thereby lowering recovery costs and mitigating performance decay under cyclic operation. Systems that combine high-affinity adsorption/reaction sites with magnetic responsiveness have demonstrated a closed-loop advantage characterized by high capacity, facile separation, and recyclability, including the ability to maintain substantial capacity after multiple cycles and regenerate under relatively mild desorption conditions.

As a widely used nanomaterial, SiO_2_ exhibits limited intrinsic CO_2_ affinity; however, its silanol-rich surface offers abundant anchoring sites for grafting or coating targeted functional groups, enabling substantial adsorption enhancement without sacrificing chemical robustness [109,110]. Along these lines, Lai et al. prepared tertiary amine functionalized nano-SiO_2_ (NS-NR_2_) via further methylation of KH540 modified nano-SiO_2_ (NS-NH_2_). The resulting material achieved high aqueous phase CO_2_ uptake, with adsorption kinetics well described by a pseudo-second-order model suggesting that chemisorption dominates the trapping pathway while cycling tests confirmed favorable regeneration stability [111].

Overall, research on nanomaterials for CO_2_ capture and separation is shifting from the single-metric pursuit of higher capture efficiency toward a more systematic paradigm of process intensification centered on structural recyclability, tunable interfacial/catalytic properties, and operational renewability. The key scientific challenge is to establish a transferable structure–interface–mass transfer–regeneration relationship, so that material-level gains can be reliably translated into system-level benefits under realistic cyclic constraints and engineering tolerances thereby enabling a scalable pathway from mechanistic validation to closed-loop deployment within CGUS.

**Table 2:** Research progress of nanomaterials in enhancing CO_2_ capture and separation.

Nanomaterials	Size	Functionalization	Key Findings	Ref
TiO_2_	25 nm	none	In a 25 wt% K_2_CO_3_ solution, TiO_2_ significantly improves the absorption efficiency by enhancing solution properties and promoting mass transfer, with an optimal absorption window appearing around 70 °C and 0.06 wt%.	[105]
Fe_3_O4 -SiO_2_	17–25 nm	KH550	Fe_3_O_4_@SiO_2_-NH_2_ significantly improved the CO_2_ capture capacity of sulfoxide-M, increasing the CO_2_ capture rate by 13.36% compared to the base solution.	[98]
TiO_2_	3 nm	EDA	Amorphous TiO_2_ nanoparticles modified with ethylenediamine showed a higher CO_2_ adsorption capacity than conventional TiO_2_ and mesoporous SiO_2_ (65 cm^3^/g at 0 °C and 100 kPa).	[112]
Silica	123.6 nm	KH540	Modified SiO_2_ exhibits low viscosity, high CO_2_ capture capacity, and good regenerability in water. The correlation coefficients show that the pseudo-second-order model agrees well with the experimental data.	[111]
Silica	12–24 nm	trimethoxyoctadecylsilane	Surface-functionalized SiO_2_ nanoparticles encapsulated in NaOH aqueous solution and dispersed in harmless silicone oil exhibit a CO_2_ capture capacity of 7.57 mmol-CO_2_/g.	[113]
Mesoporous silica	340 nm	PEHA	Diamine-functionalized mesoporous SiO_2_ exhibits an adsorption capacity of 3.62 mmol/g, high adsorption cycle stability, and high amine efficiency, along with a faster desorption rate and lower regeneration energy consumption.	[100]
MWCNTs -ZnO		APTES	The material exhibits a maximum CO_2_ adsorption capacity of 1.32 mmol/g, and the increased density of ZnO nanoparticles enhances the CO_2_ affinity under low pressure.	[99]
CNT		TEPA	When the TEPA loading reaches 75%, the maximum carbon dioxide adsorption capacity of 5 mmol/g is obtained in a gas mixture containing 10% CO_2_ and 1% H_2_O by volume.	[114]
CNT		PEI	The modified material exhibits a CO_2_ adsorption capacity of 98.8 mg/g and a good renewability of 96.2%.	[115]

### 4.2. Interface and Wettability Control: Enhancing Capture/Utilization Capabilities and Reducing Leakage Risk

During CGUS operations, the wettability and interfacial tension of the CO_2_ water rock three-phase system exert first-order control over injection pressure requirements, residual-gas trapping capacity, and the capillary sealing effectiveness of caprock. Accordingly, storage security is not governed solely by how much CO_2_ is injected, but, to a large extent, by how CO_2_ is distributed and how it migrates at the pore scale [56]. In this context, nanomaterials provide mechanistic stabilization by forming mechanically reinforced interfacial barriers, composite adsorption layers that increase interfacial viscoelasticity, strengthen foam lamellae, elevate disjoining pressure, and suppress liquid drainage, bubble coalescence, and Ostwald ripening [116].

**Figure 9:**
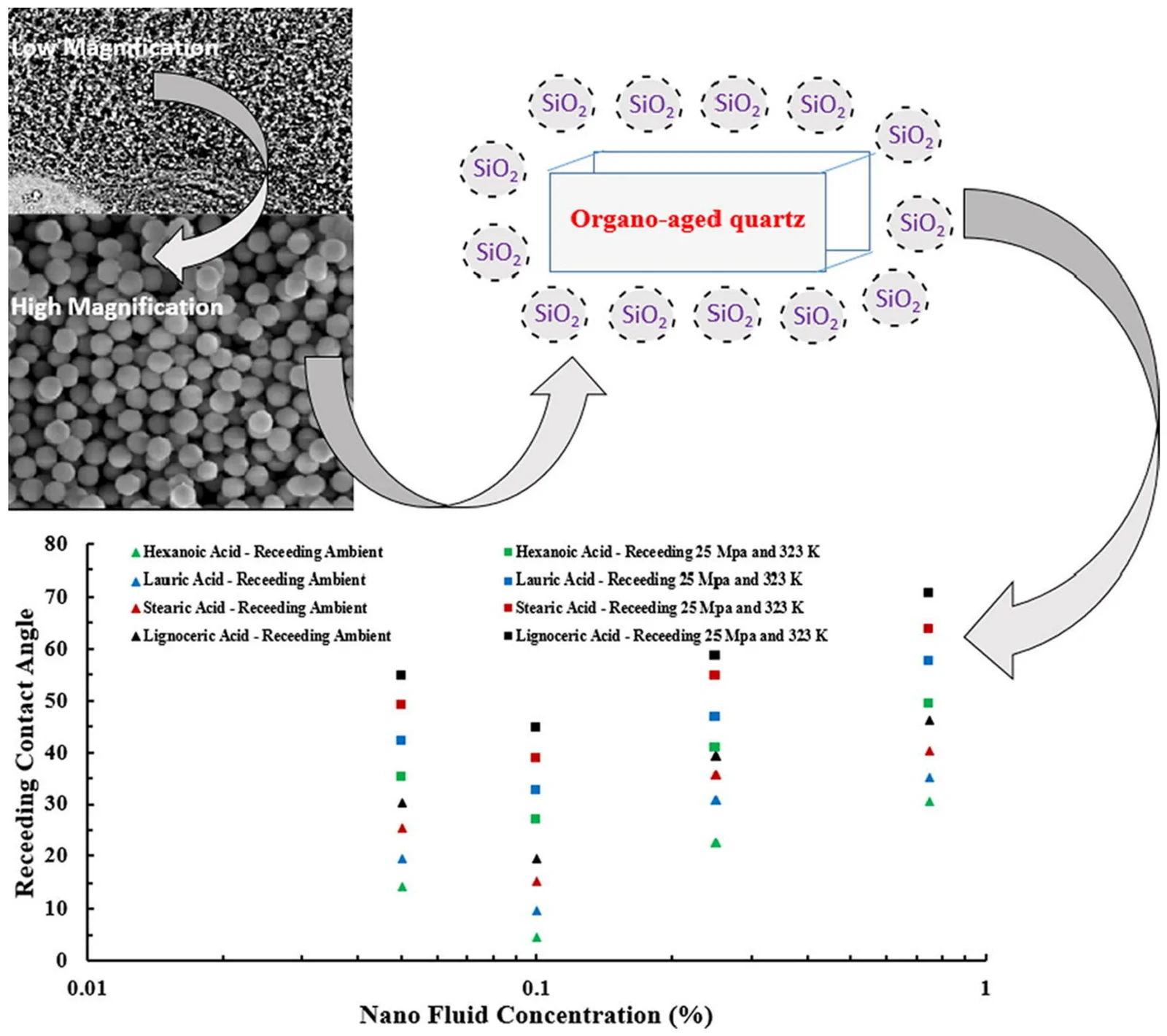
SiO_2_ nanofluids improve wettability through adsorption for CO_2_ storage [117]. Copyright 2020 Elsevier.

The engineering relevance of wetting control is particularly pronounced in relation to leakage risk. Evidence suggests that prolonged exposure to organic acids can drive sandstone surfaces from water-wet toward CO_2_-wet states, which diminishes capillary trapping and sealing capacity and consequently increases the likelihood of CO_2_ migration and leakage. Nanomaterial-based treatments offer a pathway to regulate and, in some cases, reverse such wettability shifts even on complex substrates (e.g., sandstone and shale). For example, Ali et al. reported that SiO_2_ nanofluid treatment reduced the contact angle from 123.4° to 26.9°, indicating a substantial shift toward water-wet behavior (Figure 9). Complementary EDS and SEM analyses showed that SiO_2_ nanoparticles formed a relatively uniform monolayer coating on quartz surfaces, supporting an interfacial adsorption/coating mechanism. These observations collectively suggest that SiO_2_ nanofluid treatment can restore capillary sealing and trapping capacity, thereby mitigating leakage risk and improving the reliability of geological storage in CGUS contexts [117].

**Figure 10:**
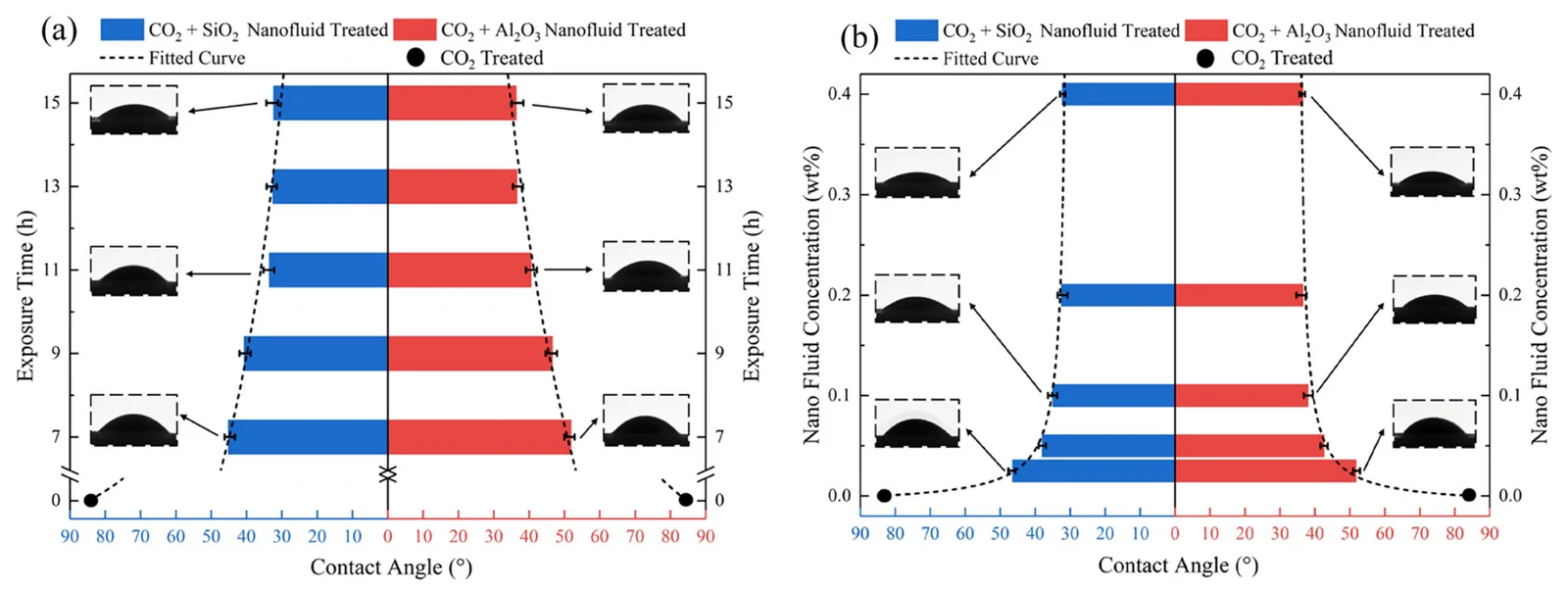
(**a**) Effect of nanofluid on contact angle as a function of aging time and (**b**) concentration [118]. Copyright 2023 Elsevier.

Ali et al. showed that Al_2_O_3_ nanoparticle suspensions alter the wettability of rock surfaces, improving CO_2_ capture promise [102]. To further reflect the influence of differences in surface charge, adsorption configuration and anti-desorption properties of different oxides on wetting reversal. Lu et al. further examined the effects of SiO_2_ and Al_2_O_3_ nanofluids on the wettability reversal of shale. Both nanofluids maintained the shale’s water-wet state, with SiO_2_ achieving a contact angle reduction from 84.6° to 32.5° after 13 h (Figure 10), and Al_2_O_3_ achieving a slightly smaller reduction (48.3%). The fundamental difference lies in the molecular weights (SiO_2_ 60.09 < Al_2_O_3_ 101.96) and distinct surface properties of the two nanoparticles, resulting in variations in molecular motion rates and adsorption capacities [118]. Table 3 shows the effect of organic acid and nanomaterials on the wettability alteration at reservoir conditions.

Introducing nanomaterials into the CO_2_ injection stream-particularly under low-salinity conditions can substantially lower the CO_2_/brine interfacial tension (IFT). Even in high-salinity CO_2_-brine systems at elevated pressure and temperature, reported IFT values can decrease from the typical 30–50 mN·m^−1^ range to approximately 22 mN·m^−1^ after nanomaterials addition [38]. Under reservoir-relevant thermodynamic conditions, nanoparticle hydrophilicity has been identified as a key determinant of IFT response. Temperature and pressure exert competing influences on interfacial interactions: increasing temperature tends to weaken the effective attractions at the CO_2_–water interface and thus elevates IFT, whereas increasing pressure generally strengthens interfacial interactions and can reduce IFT [12]. Importantly, the IFT reduction is ultimately governed by nanomaterials adsorption at the CO_2_/brine interface. Once interfacial adsorption approaches saturation, further increases in nanomaterial concentration no longer yield additional IFT decreases; the corresponding dosage thus defines a threshold concentration at which IFT converges toward an approximately constant value.

**Figure 11:**
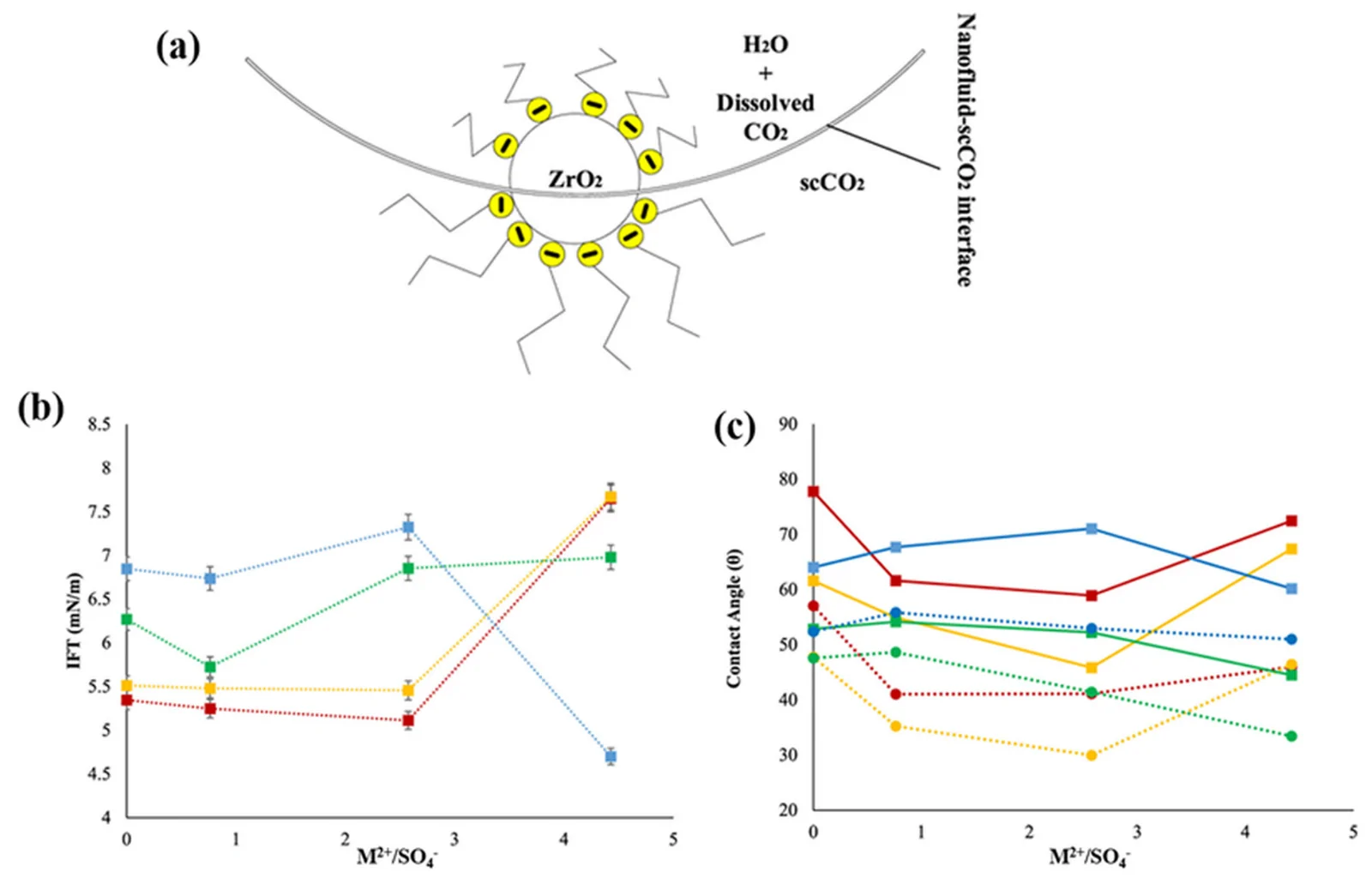
Schematic of (**a**) nanoaggregate adsorption at the nanofluid/ScCO_2_ interface, (**b**) experimental values for nanofluid/ScCO_2_ IFT against the divalent cation/sulfate ion ratio, (**c**) experimental values for LSS nanofluid/quartz contact angle [advancing (θa) and receding (θr)] against the divalent cation/sulfate ion ratio [120]. Copyright 2019 American Chemical Society.

Despite these benefits, nanoparticle-enabled interfacial and wetting regulation remains challenged by limited dispersion stability, high sensitivity to salinity/pH/divalent ions, and in some systems, a constrained IFT-reduction capability. Accumulating experimental evidence further suggests that coupling nanomaterials with surfactants can significantly enhance nanoparticle dispersion and interfacial activity in high-salinity and CO_2_-acidified brines. Under high-temperature, high-salinity conditions, such hybrid formulations can simultaneously suppress CO_2_-wetting tendencies of rock surfaces and reduce CO_2_/brine IFT [56]. For example, Jha et al. reported consistent wettability-modification behavior in rock–CO_2_–brine systems using calcite and quartz substrates with ZrO_2_ based nanofluids. The addition of commercial surfactants CTAB (0.82 mM) and SDBS (1.435 mM) stabilized the nanoparticles and produced the most pronounced reductions in both wettability and IFT (Figure 11) [119,120]. Nevertheless, while nanomaterial surfactant synergy can broaden the operational window by tuning interfacial electrical properties and restructuring adsorption layers, it also introduces additional complexity, including enhanced sensitivity to salinity and temperature and potential adsorption/retention losses that may erode injectivity or increase chemical consumption.

Mechanistically, nanomaterials achieve coupled regulation of wettability and IFT by forming persistent adsorption layers on mineral surfaces and by constructing structured interfacial films across multiphase boundaries, thereby improving capture/utilization performance and lowering leakage risk. However, subsurface interfacial states are not static; they evolve continuously in response to organic acids, salinity fluctuations, and CO_2_-induced acidification. In this sense, nanomaterials function less as a one-time modifier and more as an interfacial state regulator/restorer, whose effectiveness depends on the integrity and desorption resistance of the adsorption layer under harsh reservoir conditions. Consequently, wetting-control performance cannot be attributed solely to material type, but is governed by nanoparticle surface chemistry (e.g., functional-group identity and hydroxyl density), the nature of particle–mineral interactions, and adsorption-layer stability across variable salinity and pH. Therefore, the evaluation of different nanostructures should emphasize transferability under realistic conditions specifically, whether a uniform and durable adsorption layer can be maintained, and whether its functionality persists under CO_2_ acidification and high-salinity environments.

**Table 3:** Effect of nanomaterials on wettability of surfaces.

Nanomaterials	Size	Functionalization	Surface	Key Findings	Ref
Silica	12 nm	none	Carbonate Rocks	After adding SiO_2_, the wettability of the carbonate rock surface changed from oily to watery, and the carbonate rock became more watery. The contact angle decreased from 108° to 27.8°.	[102]
Silica	20 nm	saponin	shale	When shale substrate was treated with 0.1% SiO_2_ solution and 0.2% saponin solution, the advancing contact angle decreased by 56.34° and 56.76°, respectively. The CO_2_/NPS interfacial tension was lower than that of the CO_2_/brine interfacial tension.	[101]
MWCNT–Silica	5 nm	none	Sandstone	Nanofluids can change the wettability of rocks from oily to watery without the use of surfactants. Furthermore, the IFT can be reduced from 53.9 mN/m to 27.5 mN/m.	[121]
Fe_3_O_4_	20–25 nm	EDTA	Carbonate Rocks	The use of EDTA-modified nanoparticles can drastically alter the wettability of carbonate rocks (reducing it from 114° to 27°), and it was also found that aging temperature has a positive impact on the change in wettability.	[122]
Fe_3_O_4_	44–50 nm	SLS	Carbonate Rocks	SLS-modified nanoparticles can reduce the wettability of carbonate rocks from 140° to 22°, exhibiting higher stability in saline environments, while maintaining good recyclability.	[122]
Silica	below 100 nm	GPTMS	Quartz	The application of nanofluids effectively reduces interfacial tension and contact angle. Increased nanofluid concentration improves CO_2_ injection efficiency, with a slight upward trend observed at higher concentrations.	[123]
Al_2_O_3_	13 nm	none	Mica	The Optimum nanofluid concentration was 0.25 wt% for wettability reversal. CO_2_ wettability was reduced by alumina nanofluid. (θa = 73.5°, θr = 68.9°)	[102]
Silica	15 nm	AOS	shale	The hydrophilicity of the sample treated with nanoparticles and ScCO_2_ was increased by up to 67.93%.	[100]

### 4.3. Mobility Control and CO_2_-EOR/CGUS Synergy: Nanoparticle-Stabilized Foams

During CO_2_ injection, the inherently low viscosity and high mobility of the CO_2_ readily induce viscous fingering and gas channeling, which in turn leads to premature breakthrough, limited swept volume, and insufficient utilization of available storage space. Because foams can simultaneously increase apparent viscosity and selectively restrict flow through high-permeability pathways, they offer a practical route to achieve the coupled objectives of enhanced oil recovery, reduced ineffective cycling, and increased CO_2_ retention, and are therefore widely regarded as a key enabling technology for synergizing CO_2_-EOR with geological storage [63]. Nevertheless, conventional surfactant-based foams are prone to rapid degradation via liquid drainage, coalescence, and Ostwald ripening, particularly under harsh reservoir conditions characterized by high temperature, high salinity, oil presence, strong shear, and elevated pressure.

Recent studies suggest that nanomaterials can substantially improve foam robustness by virtue of their high desorption energy and thermochemical stability. By irreversibly (or quasi-irreversibly) adsorbing at the CO_2_–water (or CO_2_–oil–water) interface, nanomaterials form a mechanically reinforced interfacial barrier that retards bubble coalescence and suppresses coarsening dynamics [124]. Table 4 also summarizes the role of nanomaterials in influencing foam stability. Qian et al. found that CO_2_ foam stabilized with nanomaterials stored over 300% more CO_2_ than foam without nanomaterials [125], highlighting their potential to enhance both mobility control and residual trapping efficiency. As the field advances, the central challenge has shifted from demonstrating foam formation to ensuring that nanoparticle-enabled foams can retain sufficient lifetime and mobility-control strength under reservoir-relevant temperature–pressure–salinity–oil conditions, while simultaneously managing injectability constraints and minimizing risks associated with particle retention and formation damage [126].

**Figure 12:**
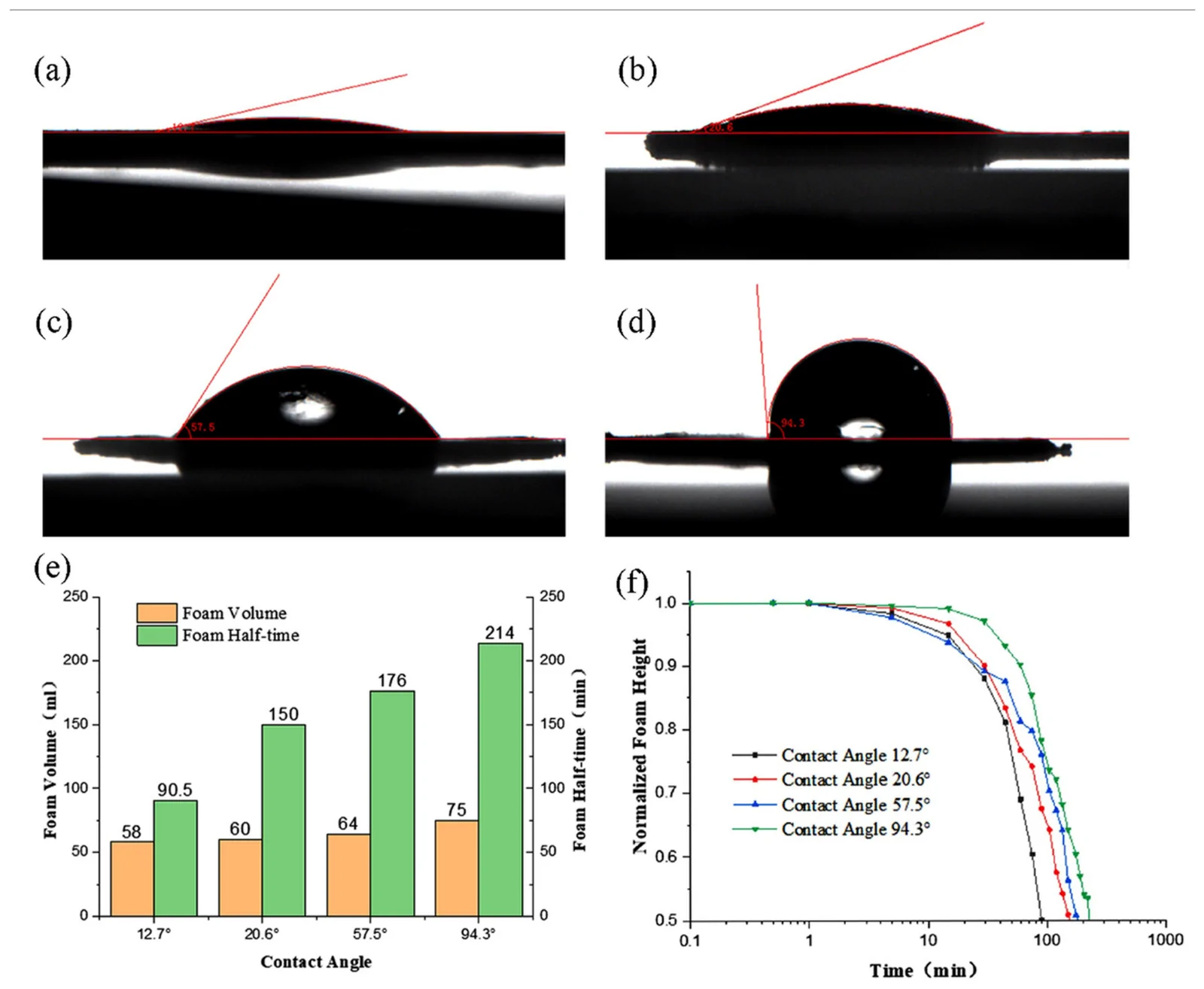
Contact angle measurements for deionized water on the surface of nanoparticles: (**a**) unmodified Fe_3_O_4_ nanoparticles, (**b**–**d**) modified Fe_3_O_4_ nanoparticles, properties of Fe_3_O_4_ nanoparticle stabilized foam with four different contact angles: (**e**) foam volume and foam half-time; (**f**) normalized foam height. [127] Copyright 2020 Elsevier.

A representative example was reported by Liu et al. who developed a Fe_3_O_4_-stabilized foam system via hydrophobic modification of Fe_3_O_4_ nanoparticles [127]. As shown in Figure 12, the modified particles achieved an optimal contact angle of 94.3°, significantly improving foam performance. The foam’s half-life reached 214 min, 2.36 times longer than the unmodified system. In displacement experiments, the Fe_3_O_4_-stabilized CO_2_ foam showed more stable pressure drops and better plugging ability in low-permeability cores. The tertiary recovery reached 10.64% Original Oil in Place (OOIP).

**Figure 13:**
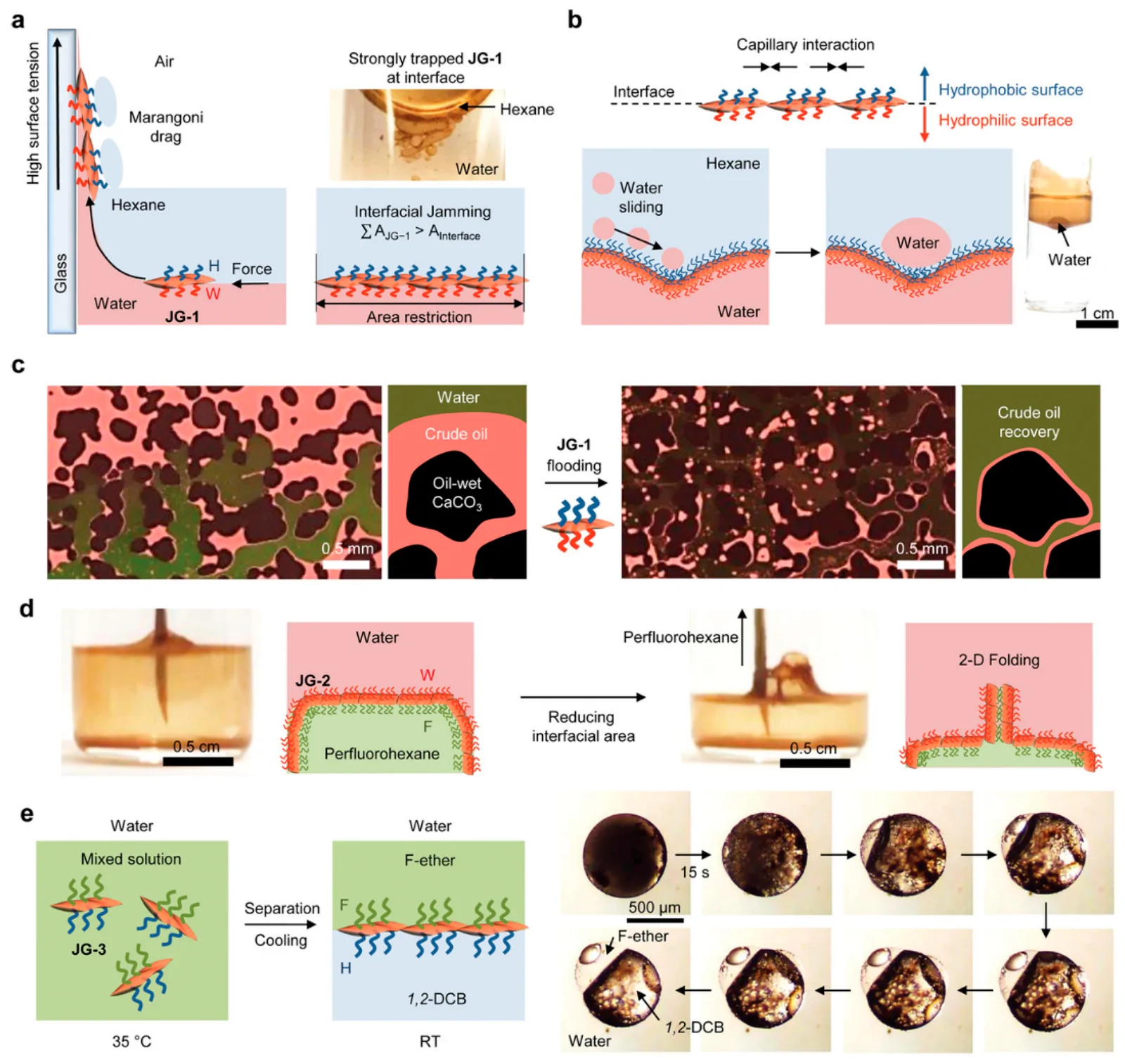
Oil Displacement Mechanism and Effectiveness of Janus Graphene [129]. (**a**) Left: JG-1 climbs along the glass vial surface from the water-hexane interface to the air-glass interface with hexane through Marangoni flow. Right: the interfacial jamming of the JG-1 surfactants is observed. (**b**) The attractive capillary interactions are expected such that the structure is connected and forms a dense edge–edge assembly. Water droplets slide on the hydrophobic surface of the 2D JG-1 assembly. The assembly of JG-1 at the interface supports a coalesced water droplet. (**c**) 2D JG-1 injection in the microfluidic system that emulates oil in a carbonate mineral formation. Left: After water (green-dyed) flooding, 39 ± 2% (determined by image processing) of crude oil (red) remained. Right: After a JG-1 water solution flooding, 25 ± 1% of crude oil remained, indicating enhanced oil removal. (**d**) When the density of JG-2 at the interface reaches the critical concentration, the JG-2 experiences an attractive force and forms bilayer assembly above the interface. (**e**) At elevated temperature 35 °C above Tc, 1,2-DCB and F-ether with JG-3 are miscible and emulsified in aqueous 0.1% Zonyl FS-300 and 0.1% sodium dodecyl sulfate. Below Tc, the mixed phase separates to create a Janus droplet in water. During cooling, JG-3 moves to the 1,2-DCB and F-ether interface.Copyright 2019 Wiley.

Two-dimensional (2D) nanomaterials are distinguished by their ability to deliver interfacial rheological reinforcement and improved aging resistance. Recent displacement studies employing nanofluids formulated with graphene-based Janus nanosheets have reported notable performance gains. Relative to conventional zero-dimensional (0D) nanoparticles, graphene-derived systems can exhibit stronger interfacial activity because their intrinsic amphiphilicity—arising from spatially distributed hydrophobic and hydrophilic domains—facilitates adsorption at multiphase interfaces and can contribute to interfacial-tension reduction. In tertiary recovery tests, graphene-based Janus nanosheet nanofluids have been reported to increase oil recovery by up to 15.2% [128]. Along similar lines, Jeon et al. prepared Janus graphene nanosheets via targeted functionalization, enabling the material to combine Pickering-type interfacial anchoring with enhanced stability of complex multiphase interfaces; compared with waterflooding, the nanosheets improved recovery by approximately 14% (Figure 13) [129]. Collectively, these results highlight the distinctive potential of 2D materials to realize high-area interfacial coverage coupled with enhanced interfacial viscoelasticity, thereby strengthening the mechanical integrity of foam films or emulsified interfaces and improving mobility-control performance.

Notably, 2D materials often rely more strongly on surface functional groups and edge/surface amphiphilic patterning to achieve stable interfacial adsorption. Consequently, their design logic should shift away from simple concentration stacking toward precise control of surface chemistry and anisotropic wetting configurations. Such a strategy can mitigate aggregation risks in high-salinity brines and improve tolerance to oil-phase interference, thereby widening the effective operating window under reservoir-relevant conditions.

These findings also reinforce a broader point: foam stability is not dictated by material category alone, but emerges from the coupled effects of particle type, surface modification, interfacial assembly, and porous-media transport. Accordingly, the development of nanoparticle-stabilized foams should be guided by the integrated interplay among interfacial reinforcement bubble-film rheology pore-blockage regulation system-level synergistic benefits, with the operating window and field-condition tolerance serving as the primary design criterion rather than material identity per se.

The literature consistently shows that CO_2_ foams suffer pronounced stability deterioration under high-temperature and high-salinity reservoir conditions, highlighting the need to identify stabilizers that simultaneously satisfy chemical robustness, environmental compatibility, foamability, and cost acceptability [130]. Nanomaterials enhance CO_2_ foam stability by reinforcing interfacial films and forming composite adsorption layers with surfactants, thereby improving foam lamella strength and suppressing liquid drainage, bubble coalescence, and Ostwald ripening [131].

Comparative studies further suggest that in ternary formulations (e.g., nanoparticle-surfactant-polymer systems), nanomaterials promote tighter interfacial packing, strengthen bubble-bubble electrostatic repulsion, and extend foam half-life [85]. Importantly, the stabilizing outcome is highly sensitive to particle wettability: moderately hydrophobic particles often favor the formation of thicker lamellae and even percolating three-dimensional interfacial/particle networks that resist rupture, whereas overly hydrophilic particles may accelerate liquid expulsion by perturbing membrane hydration structure and increasing the effective ionic strength within the lamella [132]. These observations underscore that more extreme hydrophilicity or hydrophobicity does not necessarily translate into better performance; rather, wettability should be optimized to serve the target foam-film microstructure and interfacial rheology.

**Figure 14:**
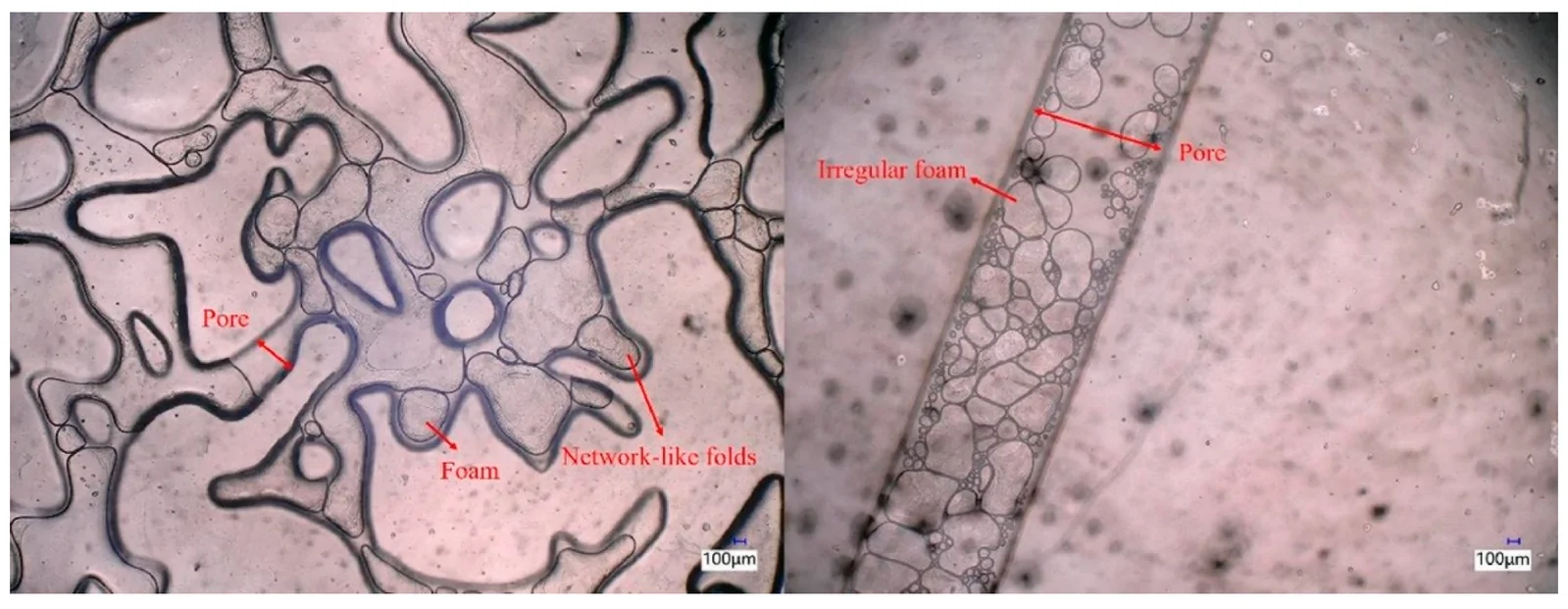
Network-like fold foams formed by SiO_2_ aerogel nanofluids and CO_2_. [25] Copyright 2022 Elsevier.

**Figure 15:**
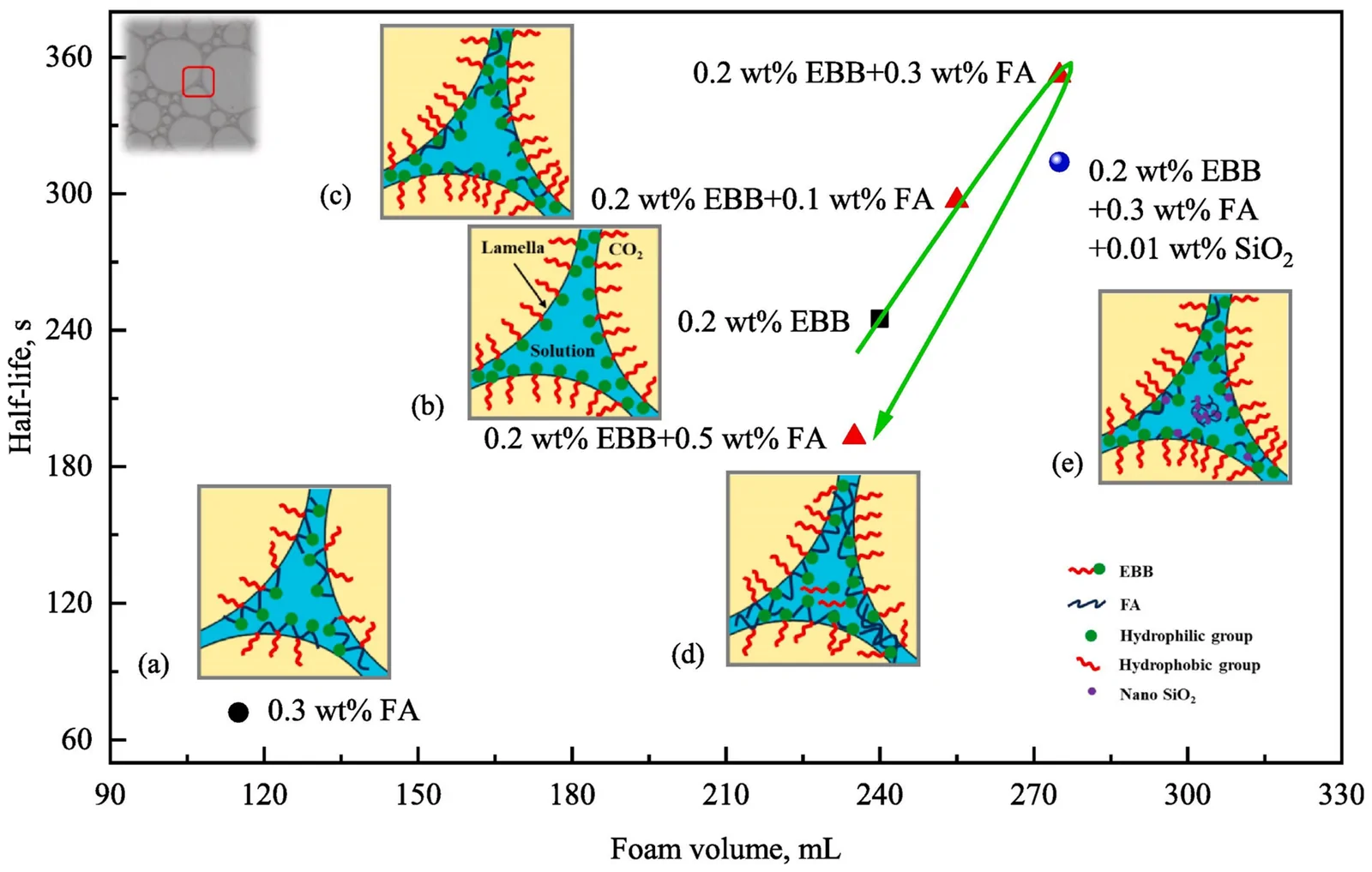
Schematic diagram of foam properties of EF and EFS foaming systems and adsorption of EBB, FA and nano-SiO_2_ molecules on the sheet [65]. (**a**) shows the schematic diagram of the arrangement of EBB molecules at the lamella; (**b**) the FA molecular chain is longer and the number of active functional groups is much lower than that of EBB molecules, the adsorption amount at the lamella is significantly reduced; (**c**) EBB molecules and FA molecular chains are interspersed and adsorbed on the lamella; (**d**) as the concentration of FA continued to increase, it would compete with EBB molecules for adsorption at the lamella, which weakened the adsorption amount of EBB molecules; (**e**) the adsorption of nano-SiO2 increases the weight of the liquid film and promotes liquid drainage, resulting in poor foaming performance. Copyright 2024 Elsevier.

To further investigate the application of the nanomaterial CO_2_ foam system in CO_2_ geological sequestration, Du et al. combined SiO_2_ aerogel with foam for oil displacement to reduce carbon emissions (Figure 14). Microscopic visualization of displacement experiments revealed that aerogel nanofluids significantly outperformed water and conventional nanofluids in CO_2_ absorption. Nanofluid displacement controlled gas flow and enhanced CO_2_ sequestration by forming stable foam, achieving a sequestration rate of 60.8% [25]. Under more complex operating conditions, the composite heat-resistant foam system (polymer surfactant FA, foaming agent EBB and SiO_2_ nanoparticles) simultaneously achieved a 13.74% increase in recovery, a 15.23% reduction in CO_2_ injection volume, and a 3.53% improvement in storage capacity (Figure 15). This establishes less injection higher synergistic benefits as a quantifiable objective [65]. This also reminds researchers that bubble designs truly tailored for CGUS should pursue quantifiable goals of less injection higher synergistic benefits, rather than merely maximizing a single static lifespan metric.

Accordingly, future evaluations should translate these design principles into reservoir-relevant metrics by linking foam lifetime with pore-scale mobility control and coupled EOR CO_2_ storage performance under realistic pressure, salinity, and temperature conditions. Within this framework, surface wettability (hydrophobicity/hydrophilicity balance), particle size (and its matching with interfacial curvature and pore structure), and interfacial charge/electrostatic compatibility should be treated as core, engineerable design parameters. Such a shift enables foam formulations to be optimized not only for stability in static tests, but for persistence, transportability, and controllable retention under realistic reservoir conditions thereby bridging laboratory feasibility with predictable field performance and scalable deployment.

**Table 4:** Effect of nanomaterials on stabilized foams.

Nanomaterials	Size	Functionalization	Key Findings	Ref
Silica	222.6 nm	APTES	Surface modification enhances the migration ability of SiO_2_ to the water–gas interface and improves foam stability. When the stabilizer is replaced from SiO_2_ to modified SiO_2_, the maximum foam half-life is extended by 33.3%.	[131]
Fe_3_O4@SiO_2_	147.252 nm	KH-304	The optimal contact angle for stable foam nanoparticles is 77.01°, with a drainage half-life of 452 s for the foam system. After foam stabilization experiments, the material can be recovered and reused.	[8]
Silica aerogel	15–25 nm	non	Nanomaterials endow the foam with exceptional thermal stability, reducing gas diffusion rates by a factor of five. Carbon dioxide capture efficiency reaches 81.23%, with recovery rates significantly increasing by 13.11%.	[124]
Amphiphilic Carbon Dots	1–2 nm	tetradecylamine	Amphiphilic carbon dots can assemble, accumulate, and stack at the CO_2_–water interface, thereby suppressing emissions, coarsening, and coalescence, significantly enhancing foam stability.	[133]
Janus silicon quantum dots	3.41 nm	TMVS	Janus silicon quantum dots generate stable microbubbles with an average diameter of 18.5 μm, maintaining excellent colloidal stability under reservoir conditions. This ultimately enhances CO_2_ sequestration efficiency by approximately 16% and increases crude oil recovery rate by about 22.3%.	[128]
Magnetic quantum dots	5.7 nm	silane-coupling ionic liquid	Demonstrates exceptional foam stability and salt resistance at ultra-low concentrations (0.005 wt%), exhibits unique defoaming properties in constant magnetic fields, and enables controllable foam behavior in high-temperature environments.	[134]
MoS_2_ nanosheets	100 nm	ODA	Modified MoS_2_ nanosheets form a synergistic system with AOS through electrostatic interactions. The nanosheets adsorb onto the foam interface to create a protective layer, significantly enhancing foam stability in high-salinity environments.	[135]
Graphene Quantum	3 nm	non	The nanofluid resulted in improved bulk foam stability due to the generation of finely structured foam of a low average bubble size (277.83 μm) and thick lamellae (98.91 μm).	[103]

### 4.4. Accelerated Mineralization: From Thermodynamics to Kinetics

Mineralization sequestration is widely regarded as the most secure long-term storage pathway. Although carbonate formation is thermodynamically favored, its realization under geological conditions is frequently limited by kinetic bottlenecks, including restricted reactant supply, transport across interfacial water films, nucleation barriers, and passivation by product layers. Within this context, nanomaterials should be positioned not merely as additives, but as tools to accelerate rate-determining steps and reconfigure reaction pathways: on the one hand, by expanding effective reaction interfacial area and introducing catalytic/promoting sites; on the other hand, by locally modulating water activity and ionic microenvironments to relax kinetic constraints and enable faster progression toward stable carbonate phases.

In settings closer to in situ sequestration where ScCO_2_, rock surfaces, and thin water films coexist mineralization often proceeds within nanoscale water films or locally confined aqueous domains, exhibiting kinetic controls that can deviate substantially from conventional bulk dissolution–precipitation frameworks. Studies using MgO nanocubes as a model system provide direct evidence that, under humid CO_2_ atmospheres, interfacial reactions mediated by nanoscale water films can generate transient precursors such as amorphous magnesium carbonate (AMC), which subsequently regulate crystalline phase evolution and overall mineralization rates. This thin water film precursor pathway implies that effective nanomaterial design parameters extend beyond surface area to include surface wettability (hydrophilicity/hydrophobicity balance) and the persistence/stability of the interfacial water film itself. Moreover, surface functionalization and particle-size effects can systematically amplify this interfacial route: MgO surfaces functionalized and treated with polar solvents have been reported to exhibit markedly enhanced carbonation rates with pronounced size dependence. The additional introduction of Si-based species can further accelerate carbonation, collectively pointing to a coupled mechanism linking surface chemistry local hydration/dehydration microenvironment nucleation and transformation pathways.

At present, CO_2_ mineralization proceeds slowly in practical systems, with the overall rate commonly constrained by the kinetically limited hydration of CO_2_. Under standard conditions, the intrinsic hydration rate constant is on the order of 10^−6^ mol·L^−1^·s^−1^, which constrains carbonate-system formation and delays downstream nucleation and growth [136]. Accordingly, the central challenge for controllable mineralization acceleration is the rational design of nano catalysts that are process-compatible with mineral formation and evolution. Such catalysts should be able to engineer the interfacial microenvironment particularly local acid–base conditions and water activity to promote CO_2_ hydration, increase effective reaction rates, and reduce kinetic barriers without inducing premature scaling or destabilizing multiphase transport. Further progress requires overcoming hydration and interfacial-transport kinetic limitations, clarifying confined mineral phase-transition pathways, and preventing passivating product layers that hinder sustained dissolution and mineralization.

**Figure 16:**
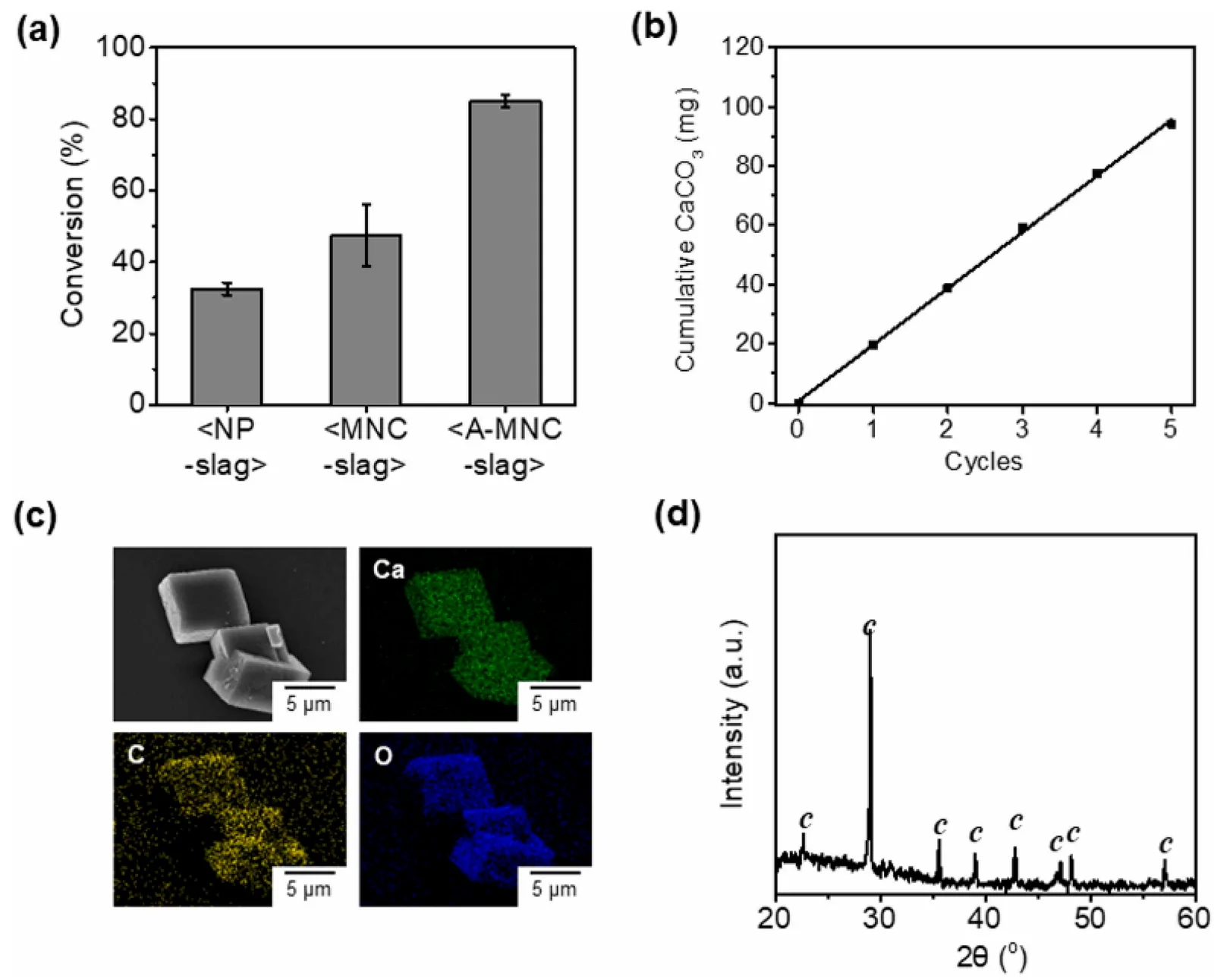
(**a**) Changes in the conversion efficiency into CaCO_3_, (**b**) The cumulative amount of precipitated CaCO_3_ produced over five cycles using A-MNCs. (**c**) SEM image and EDS mapping of the precipitated CaCO_3_ after mineralization in A-MNC, (**d**) XRD patterns of the precipitated CaCO_3_ after mineralization in A-MNC [137], Copyright 2024 Elsevier.

Han et al. developed amine-functionalized magnetic nanoparticle clusters (A-MNCs), composed of Fe_3_O_4_ nanoparticles, to improve carbon mineralization efficiency. These clusters enable CO_2_ conversion into stable carbonate minerals. The rate of CO_2_ dissolution was enhanced in the presence of A-MNCs, mostly due to physical effects such as grazing and hydrodynamic effects that improved mass transfer between the solution and CO_2_ bubbles. In addition, A-MNCs promoted carbon mineralization in a NaOH solution containing Ca^2+^ by providing an additional mineralization route via carbamate formation, resulting in a threefold increase in efficiency to 81% (Figure 16) [137]. Zhao et al. has developed a thermally stable, biomimetic carbonic anhydrase nanogel. This material accelerates carbon dioxide mineralization while simultaneously producing light hydrocarbons such as ethane, ethylene, and propane [138]. Its contribution extends beyond enhancing any single transient metric; it integrates reaction intensification solid–liquid separation recycling into a single closed-loop process, aligning with the engineering logic of mineralization coupled with resource/material pathways emphasized in the literature on mineralization pathways.

Carbonic anhydrase (CA) is widely recognized as a key catalytic unit for reactive capture because it can dramatically accelerate CO_2_ hydration/dehydration. However, its practical deployment has long been limited by insufficient operational stability and poor reusability, motivating growing interest in immobilizing CA onto solid carriers particularly nanomaterials to overcome these engineering bottlenecks. Immobilization can preserve the intrinsic hydration-catalysis function while enabling performance optimization through carrier morphology, interfacial microenvironment, and immobilization chemistry, thereby improving cyclic durability under process-relevant conditions.

**Figure 17:**
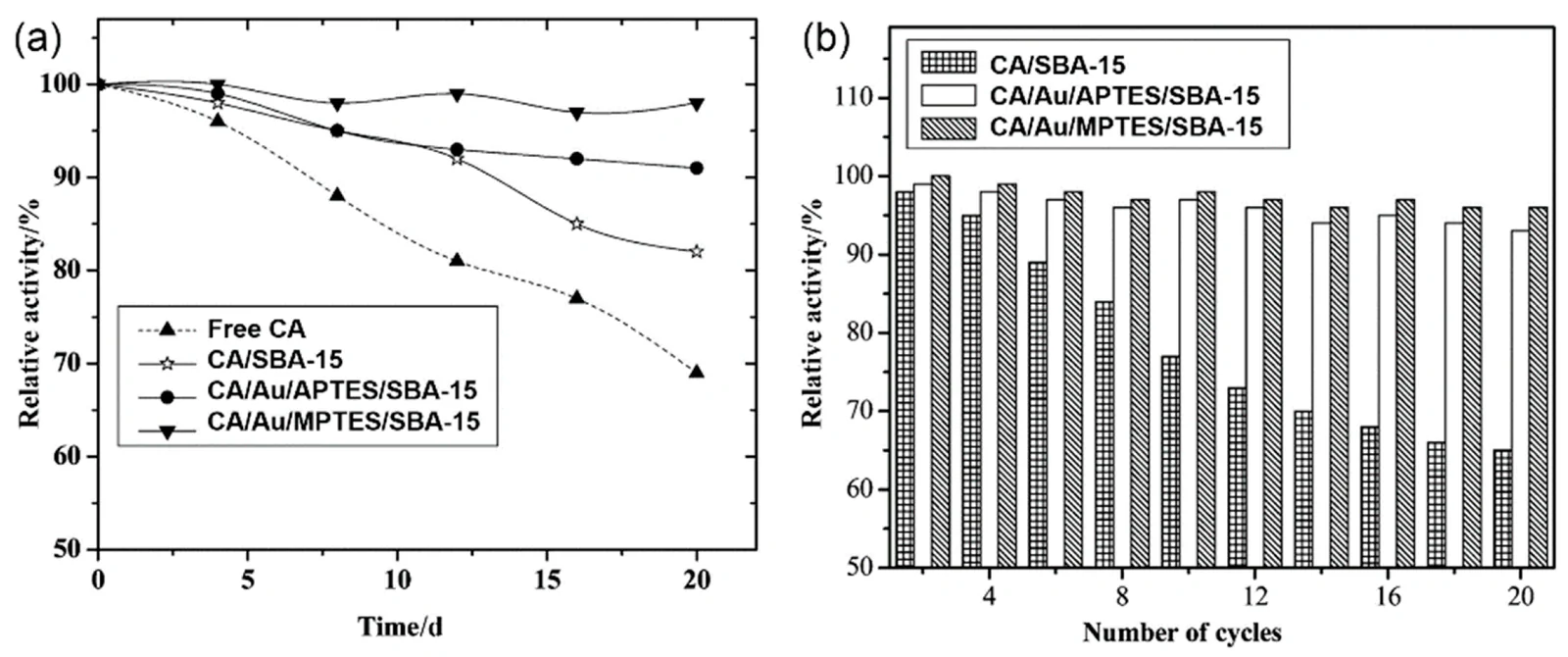
(**a**) The reusability of free and immobilized CA for hydrolysis of p-NPA, (**b**) The storage stability of free and immobilized CA for hydrolysis of p-NPA [139], Copyright 2011 American Chemical Society.

**Figure 18:**
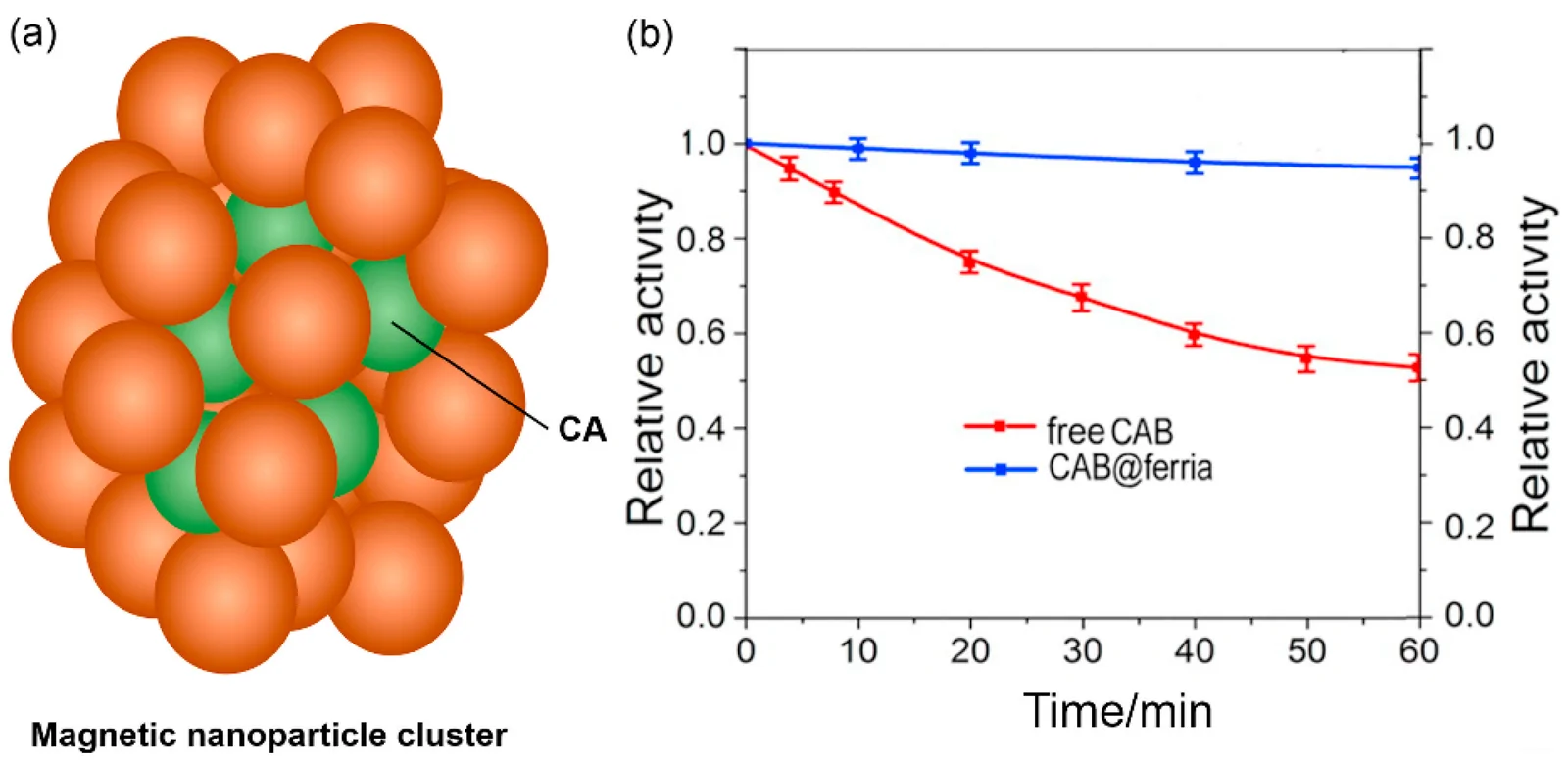
(**a**) Schematic illustration of CA immobilization on magnetic nanoparticle cluster. (**b**) The activity of free CA and immobilized CA after exposure to a temperature of 65 °C for 1 h [140], Copyright 2016 American Chemical Society.

Vinoba et al. immobilized the catalyst on gold nanoparticles assembled on the surface of amino/thiol-modified mesoporous silica (SBA-15). The catalytic efficiency of CA/Au/MPTES/SBA-15 and CA/Au/APTES/SBA-15 was nearly identical to that of free CA. Furthermore, CA/Au/MPTES/SBA-15 retained 96% of its initial activity after 20 cycles, whereas CA/Au/APTES/SBA-15 and CA/SBA-15 exhibited activity declines of 7% and 21%, respectively. Hydrolysis experiments were conducted over 20 days at 25 °C with 4-day intervals for both free and immobilized CA. The residual activities of CA/SBA-15, CA/Au/APTES/SBA-15, and CA/Au/MPTES/SBA-15 retained 82%, 91%, and 98% of their initial activity, respectively, while the catalytic activity of free CA significantly decreased to only 31% of its initial activity (Figure 17) [139]. Vinogradov et al. prepared magnetic enzyme composites using CA encapsulated within a magnetite matrix synthesized via the sol–gel method under neutral pH conditions (Figure 18). In this system, the encapsulated CA catalyzed the decomposition of p-NPA at 90 °C, while free CA underwent complete denaturation at 70 °C. After one hour of heat treatment at 65 °C, free CA exhibited a 45% loss in activity, whereas immobilized CA retained 95% of its catalytic efficiency. Furthermore, thermal stability improved by an order of magnitude: the degradation rate of immobilized CA was only 0.08%, compared to 0.9% for free CA [140]. Taken together, these studies indicate that nanoparticle-enabled immobilization can simultaneously enhance CA stability, preserve catalytic activity, and improve recyclability thereby providing a practical route to link catalytic hydration-CO_2_ uptake-mineralization into a controllable and experimentally verifiable process chain.

In summary, while substantial theoretical advances and laboratory-scale demonstrations have established the feasibility of nanomaterial-enabled mineralization acceleration, several mechanistic gaps still limit reliable translation to subsurface deployment. In particular, it remains necessary to resolve the nucleation and crystallization mechanisms of carbonates within nanoconfined water films, as well as to clarify mineralization pathways associated with wet supercritical CO_2_ environments. Addressing these questions requires a multiscale framework that links molecular-level interfacial processes to observable kinetic behavior. In this regard, coupling quantum-chemical calculations with molecular dynamics simulations provides a promising route to uncover the governing mechanisms of carbonation in CO_2_–water systems, including hydration/speciation dynamics, interfacial ion pairing, and precursor-to-crystal transformation pathways. In parallel, advanced experimental tools such as atomic force microscopy can be leveraged to probe supercritical CO_2_–mineral interactions at relevant interfaces and to directly interrogate the conditions that regulate carbonate nucleation, growth, and morphology. Together, these nanoscale insights will provide the mechanistic foundation for developing CO_2_ mineralization strategies that are not only fast, but also operationally safe and durable, thereby supporting genuinely long-term (century-scale) sequestration.

## 5. Challenges and Prospects for Large-Scale Promotion and Application

Although various nanomaterials demonstrate unique advantages in the CGUS process, the transformation from laboratory research to large-scale engineering applications still faces numerous challenges. These challenges span multiple dimensions, including theory, technology, economics, and safety. A systematic analysis of these limiting factors, along with anticipating future development directions, is crucial for advancing the industrialization of nano-material-enhanced CGUS technology. Meanwhile, Table 5 summarizes the key challenges and future research directions for nanomaterial enhanced CGUS.

(1) Material Stability and Service Life: In the high-temperature, high-pressure, highly mineralized environment with the coexistence of multiple components in the formation, the physical and chemical stability of nanomaterials faces significant challenges. Metal oxide nanomaterials (e.g., Fe_3_O_4_, MgO) may undergo redox reactions, dissolution, or crystal phase transformations. Carbon-based materials (such as graphene oxide) may experience structural delamination or functional group degradation under high-temperature hydrothermal conditions. Surface modifiers (such as silane coupling agents) may hydrolyze and detach, leading to nanoparticle agglomeration and deactivation [85,141,142]. These degradation behaviors can significantly reduce the CO_2_ adsorption capacity, catalytic activity, and foam stability, thereby affecting the long-term effectiveness of the storage process.

(2) Scalable Production and Cost: Currently, the synthesis of high-performance nanomaterials is limited to laboratory scale. Scaling up production presents challenges such as low yield, high energy consumption, and significant batch-to-batch variability. For instance, graphene produced by chemical vapor deposition (CVD) is costly [143], while the hydrothermal synthesis of monodisperse metal oxide nanoparticles has limited capacity [144]. The functionalization modification process often involves organic solvents, and the subsequent processing is complex and expensive. These factors present dual challenges to large-scale application in terms of economic viability and production efficiency.

(3) Environmental and Ecological Risks: Nanoparticles injected underground may migrate through fractures or pores into non-target strata, or even enter aquifers. Their environmental behavior, such as aggregation-sedimentation and colloidal stability, is influenced by multiple factors, including formation water chemistry, rock properties, and microbial activity [116]. Currently, there is a lack of long-term field monitoring data. Certain metal nanoparticles (such as CuO, ZnO) may exert toxic effects on soil microbial communities [145,146]. Hydrophobic-modified nanoparticles may adsorb organic pollutants, enhancing their mobility. However, existing risk assessment models are still unable to accurately predict the long-term fate and ecological impact of nanomaterials in strata [41]. Therefore, a more comprehensive environmental behavior research and assessment system is needed.

(4) Lack of Multi-Scale Mechanism Understanding and Models: The synergistic mechanism of nanomaterials between the pore scale (nm-μm) and the reservoir scale (m-km) is complex. Existing numerical models still face challenges in fully coupling nano-effects. For instance, fundamental data remains scarce regarding the adsorption behavior of nanoparticles at the CO_2_-brine-crude oil three-phase interface, the interfacial reaction kinetics of nanocatalysts, and the migration patterns of nanofoam within heterogeneous pores [20,21,147]. The lack of cross-scale prediction tools has led to the design of nano-enhanced CGUS schemes relying more on experience, making the optimization process significantly challenging. Therefore, there is an urgent need to establish a unified multi-scale mechanistic model to better guide the application of nanomaterials in CGUS technology.

Despite the severity of the aforementioned challenges, many bottlenecks are gradually being overcome through interdisciplinary collaboration and technological innovation. Recent advances in material design, process integration, and risk assessment have provided new approaches and development opportunities for the large-scale application of nanomaterials in CCUS, paving the way for more efficient and sustainable CO_2_ capture, utilization, and storage systems. These advancements not only address key technical limitations but also enhance the scalability and environmental viability of CCUS technologies, positioning them as critical components in the global effort to mitigate climate change.

(5) Multifunctional Composite Nanomaterials Design: By constructing composite materials that integrate “adsorption-catalysis-storage” (such as metal oxide-carbon-based hybrid materials and SiO_2_-based composite particles), it is possible to achieve efficient capture and in situ mineralization of CO_2_ [20,148]. This integrated design not only enhances CO_2_ capture efficiency but also enables the captured carbon dioxide to rapidly transform into stable mineral forms within the reservoir, further strengthening storage effectiveness. Additionally, the development of smart materials that respond to changes in pH, temperature, and CO_2_ concentration allows for on-demand regulation of the adsorption–desorption process [149,150]. This can effectively reduce the regeneration energy consumption of materials and improve the overall economic efficiency and sustainability of CGUS technology.

(6) Developing green and low-carbon nanomaterial preparation technology is the key to promoting the industrial application of nanomaterials. For example, employing low-energy processes such as biomimetic templates (e.g., diatomaceous earth-derived SiO_2_), waste resource utilization routes (e.g., extracting Al_2_O_3_ nanoparticles from fly ash), and microwave-assisted synthesis can significantly reduce production costs and environmental pollution [126,151,152,153]. Furthermore, establishing a life-cycle assessment system for nanomaterials in CGUS applications to clarify their carbon reduction potential and sustainability will help achieve the dual objectives of environmental and economic benefits.

(7) Advancing Multi-Scale Intelligent Computing and AI-Assisted Design: The combination of molecular dynamics (MD) simulations, density functional theory (DFT), and studies on interfacial reaction procedure represents a promising approach to advancing nanomaterials in CGUS. The application of the pore network model (PNM) to predict the seepage behavior of nanofluids provides a theoretical basis for the design and optimization of nanomaterials [9,130,154,155]. By leveraging machine learning (ML) technology to optimize the structure and injection strategy of nanomaterials, their capability in the CGUS process can be significantly enhanced [156]. Furthermore, the establishment of a “material-fluid-formation” digital system enables precise design and dynamic control of nano-reinforced CGUS processes, offering robust support for future engineering applications [157].

**Table 5:** Key challenges and future research directions for nanomaterial-enhanced CGUS.

Aspect	Key Limitations	Potential Impacts on CGUS	Future Research Directions
Material stability and service life	Nanomaterials may undergo aggregation, dissolution, redox reactions, phase transformation, or surface-modifier degradation under high-temperature, high-pressure, and high-salinity reservoir conditions	Loss of CO2 adsorption capacity, catalytic activity, foam stability, and long-term interfacial regulation	Develop thermally stable, salt-tolerant, and chemically robust nanomaterials with durable surface functionalization.
Scalable production and cost	Current synthesis routes often suffer from low yield, high energy demand, batch variability, costly precursors, and complex post-treatment processes	Limited economic feasibility and poor reproducibility during large-scale application.	Establish low-cost, high-yield, and reproducible synthesis routes, including continuous production and solvent-reduced functionalization strategies.
Environmental and ecological risks	Injected nanoparticles may migrate into non-target formations or aquifers, and their long-term fate, toxicity, and interactions with pollutants or microorganisms remain insufficiently understood	Potential ecological risk, regulatory uncertainty, and reduced public acceptance.	Build long-term field-monitoring frameworks, nanoparticle fate models, and life-cycle environmental risk assessment systems.
Multi-scale mechanisms and predictive models	The coupling among nanoparticle transport, adsorption, interfacial reactions, foam migration, pore blockage, and reservoir-scale flow remains poorly quantified	Field design still relies heavily on empirical optimization, limiting predictability and transferability.	Develop unified multi-scale models integrating molecular simulation, pore-network modeling, reservoir simulation, and experimental validation.
Multifunctional material design	Single-function nanomaterials may not simultaneously satisfy adsorption, catalysis, mobility control, and storage-security requirements	Limited system-level efficiency in complex CGUS environments.	Design multifunctional composite nanomaterials integrating adsorption, catalytic mineralization, foam stabilization, and storage enhancement.
Green preparation and life-cycle sustainability	Some preparation routes consume large amounts of energy, solvents, or high-purity feedstocks	The environmental benefit of nanomaterial-enhanced CGUS may be weakened by upstream production impacts.	Develop green synthesis routes based on waste resources, biomimetic templates, microwave-assisted processes, and full life-cycle assessment.
AI-assisted design and digital optimization	Material screening, injection-strategy optimization, and reservoir adaptation remain time-consuming and data-intensive	Slow translation from laboratory discovery to engineering design.	Combine DFT, molecular dynamics, pore-network models, machine learning, and “material–fluid–formation” digital systems for predictive design and dynamic control.

## 6. Conclusions

This review highlights nanomaterials as a distinctive enabling platform for CO_2_ geological utilization and storage (CGUS), not merely as conventional additives but as interfacial and reactive regulators capable of linking pore-scale processes with reservoir-scale performance. Their novelty lies in the ability to simultaneously modulate wettability, interfacial tension, foam and emulsion stability, CO_2_ mobility, and mineralization pathways, thereby addressing several coupled bottlenecks in CO_2_ storage security, CO_2_ utilization efficiency, and enhanced oil recovery. A key contribution of this review is the integration of nanomaterial functions across different CGUS scenarios, including CO_2_-EOR, foam-assisted mobility control, dissolution trapping, and mineral trapping. Rather than treating these processes separately, this review emphasizes their shared dependence on interfacial behavior, nanoparticle transport, reaction kinetics, and long-term reservoir compatibility. This perspective provides a more unified framework for understanding how nanomaterials can contribute to both short-term operational efficiency and long-term CO_2_ immobilization. Future research should move beyond demonstrating isolated material performance and focus on reservoir-relevant system design. Priority should be given to nanomaterials with controllable dispersion, predictable transport, durable interfacial activity, and environmentally acceptable long-term behavior under high-temperature, high-pressure, and high-salinity conditions. Particular attention should be paid to multifunctional nanomaterials that can couple mobility control with mineralization enhancement, as well as to green, scalable, and low-carbon synthesis routes that do not compromise the overall climate benefit of CGUS. Another important direction is the development of predictive design tools that integrate molecular simulation, pore-scale visualization, reservoir modeling, machine learning, and field monitoring. Such a “material-fluid-formation” framework would enable more accurate optimization of nanomaterial structure, dosage, injection mode, and reservoir adaptability. Overall, the future value of nanomaterials in CGUS will depend not only on their intrinsic physicochemical properties but also on whether they can be engineered into safe, scalable, and economically viable subsurface processes for long-term CO_2_ management.

## Figures and Tables

**Table 1:** Comparison of the advantages and disadvantages of different modification methods.

Modification Type	Modification Method	Advantage	Disadvantage
Physical Methods	Heat treatment and mechanical modification	Economical and simple, improved dispersibility	High temperature control requirements, promise particle damage
	Physical adsorption method	Simple and efficient, with good reversibility and no damage to the structure of the particles themselves.	Limited applicability, poor stability
	Physical coating method	Improve dispersibility and simplify operation	Limited encapsulation efficiency, potential impact on particle activity, and difficulty in recovery and reuse
Chemical Methods	Esterification modification	Low cost, easy to operate	Harsh reaction conditions, byproduct issues, poor stability
	Surface grafting modification	High controllability and excellent performance complementarity	Limited modifying effect, steric hindrance influence
	Coupling modification	Enhance the compatibility between particles and matrix, improve surface functionalization ability, and be widely applicable	High consumption of coupling agents, demanding coupling reaction conditions, and relatively high cost of coupling agents

## Data Availability

All data generated or analyzed during this study are contained within the article.

## Abbreviations

CCUS	Carbon Capture Utilization and Storage
CGUS	Carbon Geological Utilization and Storage
EOR	Enhanced Oil Recovery
MMP	Minimum Miscibility Pressure
ΔG	Gibbs Free Energy
ΔH	Enthalpy
ΔS	Entropy
γ	surface free energy of the solid–liquid interface
*V_LW_*(*h*)	the Lifshitz–van der Waals contribution
*V_EDL_*(*h*)	the double electron shell contribution
*V_AB_*(*h*)	the Lewis acid–base contribution
SDS	Sodium Dodecyl Sulfate
CTAB	Cetyltrimethylammonium Bromide
PEG	Polyethylene Glycol
APTES	γ-Aminopropyltriethoxysilane
OTES	Triethoxyoctylsilane
AA	Acrylic Acid
MMA	Methyl Methacrylate
GO	Graphene Oxide
TEPA	tetraethylenepentamine
